# Computational study of the mechanism and selectivity of [3 + 2] cycloaddition reactions between nitrone and carbodiimide leading to the formation of anticancer 1,2,4-oxadiazolidine compounds from a MEDT perspective

**DOI:** 10.1038/s41598-026-47611-6

**Published:** 2026-04-10

**Authors:** Moulay Driss Mellaoui, Mohamed Moussaoui, Soukayna Baammi, Aaziz Jmiai, Rachid Benhida, Souad El Issami, Hanane Zejli, Rachid Daoud

**Affiliations:** 1 Faculty of Sciences Applied Physical Chemistry Laboratory , Ibn Zohr University , B. P. 8106 Cité Dakhla, Agadir, Morocco; 2 College of Chemical Sciences and Engineering (CCSE) Chemical and Biochemical Sciences (CBS) , Mohammed VI Polytechnic University (UM6P) , 43150 Benguerir, Morocco; 3 Institut de Chimie de Nice (ICN) UMR CNRS 7272 , Université Côte d’Azur , Provence-Alpes- Côte d’Azur, Nice, 06108 France

**Keywords:** DFT, [2π + 3π] cycloaddition, 1,2,4-oxadiazolidine, MEDT, ADMET, Docking molecular, Cancer, Chemical biology, Chemistry, Computational biology and bioinformatics, Drug discovery

## Abstract

This study explores the reactivity and pharmaceutical potential of a novel anticancer compound, 1,2,4-oxadiazolidine, synthesized through the [3 + 2] cycloaddition (32CA) reaction between nitrone (1a) and carbodiimide (2a). The mechanism, investigated using Density Functional Theory (DFT) with the B3LYP-D3 functional and the 6-311 + + G(2d,2p) basis set, reveals a polar, regioselective process involving two transition states (TS1 and TS2), with a thermodynamic preference for the ortho isomer, consistent with experimental findings. Molecular docking studies with the EGFR protein (PDB: 4HJO) indicate that compound P1 binds with a higher affinity (-8.8 kcal/mol) compared to the reference drug Erlotinib. Furthermore, molecular dynamics (MD) simulations over 100 ns demonstrate that the P1-EGFR complex exhibits greater stability and lower fluctuation, further supporting its promising interactions with the target protein. In silico ADMET analysis reveals excellent oral absorption, low toxicity, and a favorable pharmacokinetic profile. These findings comprehensively validate P1’s reactivity, affinity, and stability, positioning it as a promising anticancer drug candidate.

**Supplementary Information:**

The online version contains supplementary material available at 10.1038/s41598-026-47611-6.

## Introduction

Cycloaddition reactions are fundamental tools in organic chemistry for the highly efficient and fast synthesis of five-membered heterocyclic compounds^1,2^. The cycloaddition between nitrones, designed to act as 1,3-dipoles, and carbodiimides, acting as dipolarophiles, hold great promise for the synthesis of highly functionalized nitrogen structures^3^. These reactions generate 1,2,4-oxadiazolidine - or oxadiazole-type rings, which can then be transformed into other bioactive heterocycles^4–6^. The products of cycloaddition are structurally diverse and rich in heteroatoms (notably N and O), so they are particularly attractive for the design of new therapeutic agents^7–9^.

These structures are considered privileged scaffolds in drug design from a pharmacological perspective, as they can mimic key non-covalent interactions with biological targets, such as enzymes, receptors, or nucleic acids. For example, several derivatives containing five-membered nitrogen rings have shown antiviral^10^, antibacterial^11^, antifungal or anticancer activity^12,13^, and often serve as a starting point for the development of optimized drug candidates. On a theoretical level, extensive research based on Molecular Electron Density Theory (MEDT) has been conducted to explain reaction mechanisms., as well as aspects of regioselectivity and stereoselectivity in cycloaddition reactions^14–17^. Initially introduced in 2016 by Domingo^18^, MEDT is based on the principle that a molecule’s reactivity does not depend on interactions between molecular orbitals, as traditional theory suggested, but rather on the ability of its electron density to evolve during the reaction. This approach, in continuity with the foundations of Density Functional Theory (DFT)^19,20^, has deepened our understanding of reaction mechanisms in organic chemistry^21–24^, offering a theoretical explanation for experimental observations.

The MEDT theory classifies [3 + 2] cycloaddition reaction (32CA) mechanisms into four types according to the electronic nature of the three-atom component (CAT): pseudo-diradical, pseudo-(mono)radical, carbenoid, and zwitterionic^25,26^. This classification is based on the number of electrons present in the monosynaptic basins. The order of creasing reactivity is: zw < cb< pmr < pdr. In contrast, zwitterionic mechanisms often require external activation to react effectively. This approach provides a better understanding of the electronic nature of the reactions and related kinetic behavior.

**Scheme 1:**
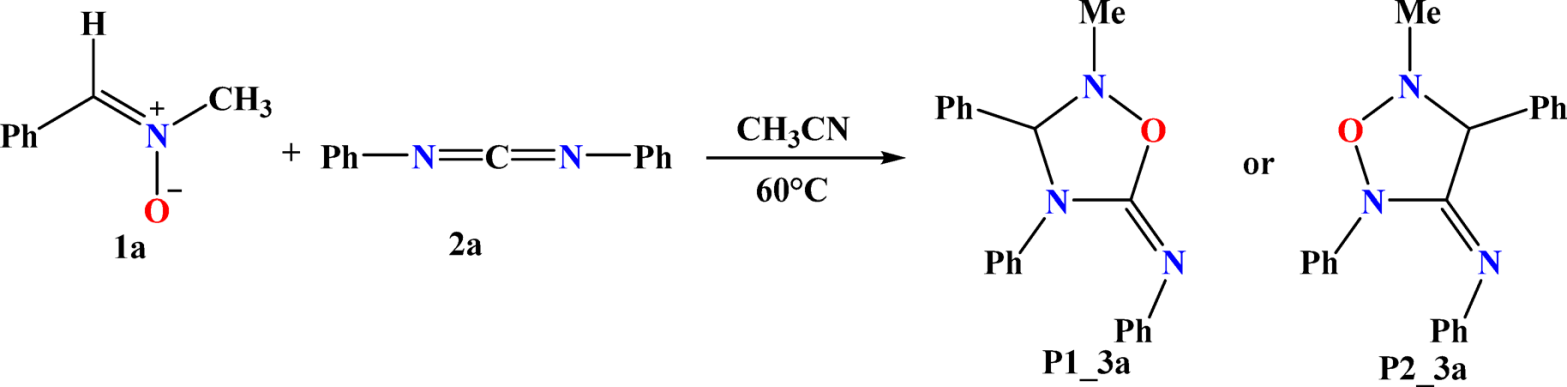
32CA reactions of reactivity of nitrone (1a) with carbodiimide (2a).

In this study, we analyzed the 32CA between nitrone (1a) and carbodiimide (2a), resulting in the formation of 1,2,4-oxadiazolidines (P1_3a or P2_3a), as reported by Chen et al.^27^. This cycloaddition exhibits high regioselectivity (see Scheme 1).

## Computational methods

In this study, all stationary points (reactants, products, and transition states) were optimized and visualized using GaussView 6.0^28^ and ChemCraft 1.8 software^29^. Comprehensive geometric optimization of the stationary points was carried out using Gaussian 16^30^, using different quantum chemical methods such as B3LYP, with and without GD3 dispersion correction^31,32^, MPWB1K and ωB97XD^33,34^. Electronic energy calculations of the DLPNO-CCSD(T)/cc-pVTZ^35–37^ type were performed using the ORCA version 6.0.1 program^38–40^, to obtain more accurate energies.

Calculations were performed using different bases, including 6-31G(d), 6–311 + + G(2d,2p)^41–43^, covering the double-zeta and triple-zeta levels. Harmonic frequency calculations were then performed to confirm the nature of the stationary points: reactants and products must have only absolute frequencies (positive eigenvalues of the Hessian matrix), while transition states must possess a single imaginary frequency, characteristic of the transition between reactants and products. To confirm that each transition state actually connects the corresponding reactants and products, IRC (Intrinsic Reaction Coordinate) calculations were carried out^44^. The effect of solvent was taken into account using the IEFPCM solvation model, with acetonitrile as solvent. The expressions for the electrophilic +() and nucleophilic -() Parr functions derived from the spin density distribution are expressed as follows:

$$\:{P}^{-}\left(r\right)=\:{\rho\:}_{s}^{rc}\left(r\right)\:\:\:\:For\:electrophilic\:attacks$$

$$\:{P}^{+}\left(r\right)=\:{\rho\:}_{s}^{ra}\left(r\right)\:\:\:\:For\:nucleophilic\:attacks$$

where $\:{\rho\:}_{s}^{rc}\left(r\right)\:$is the ASD of the radical cation, and $\:{\rho\:}_{s}^{ra}\left(r\right)\:$is the ASD of the radical anion. Each ASD condensed at the different atoms of the radical cation and radical anion provides our local nucleophilic $\:{P}_{k}^{-}$and electrophilic $\:{P}_{k}^{+}$ Parr functions of the neutral system.

Electronic properties such as electronic localization functions (ELF)^45,46^, non-covalent interactions (NCI) and molecular electrostatic potential (ESP) were analyzed using the Multiwfn program^47^, and visualizations were generated via VMD^48^.

## Results and discussion

### A molecular electron density theory study for [3 + 2] cycloaddition reactions of nitrone 1a and carbodiimide 2a

#### Analysis of the ELF topology of the reactants

The Electron Localization Function (ELF) is a method that utilizes electron density to visualize and identify the locations of electron pairs within a molecule. It helps pinpoint binding regions, non-bonding doublets, and areas of electron delocalization. High ELF values suggest strong electron localization, such as covalent bonds or free doublets, while low values point to delocalized regions or weak interactions.

**Fig. 1:**
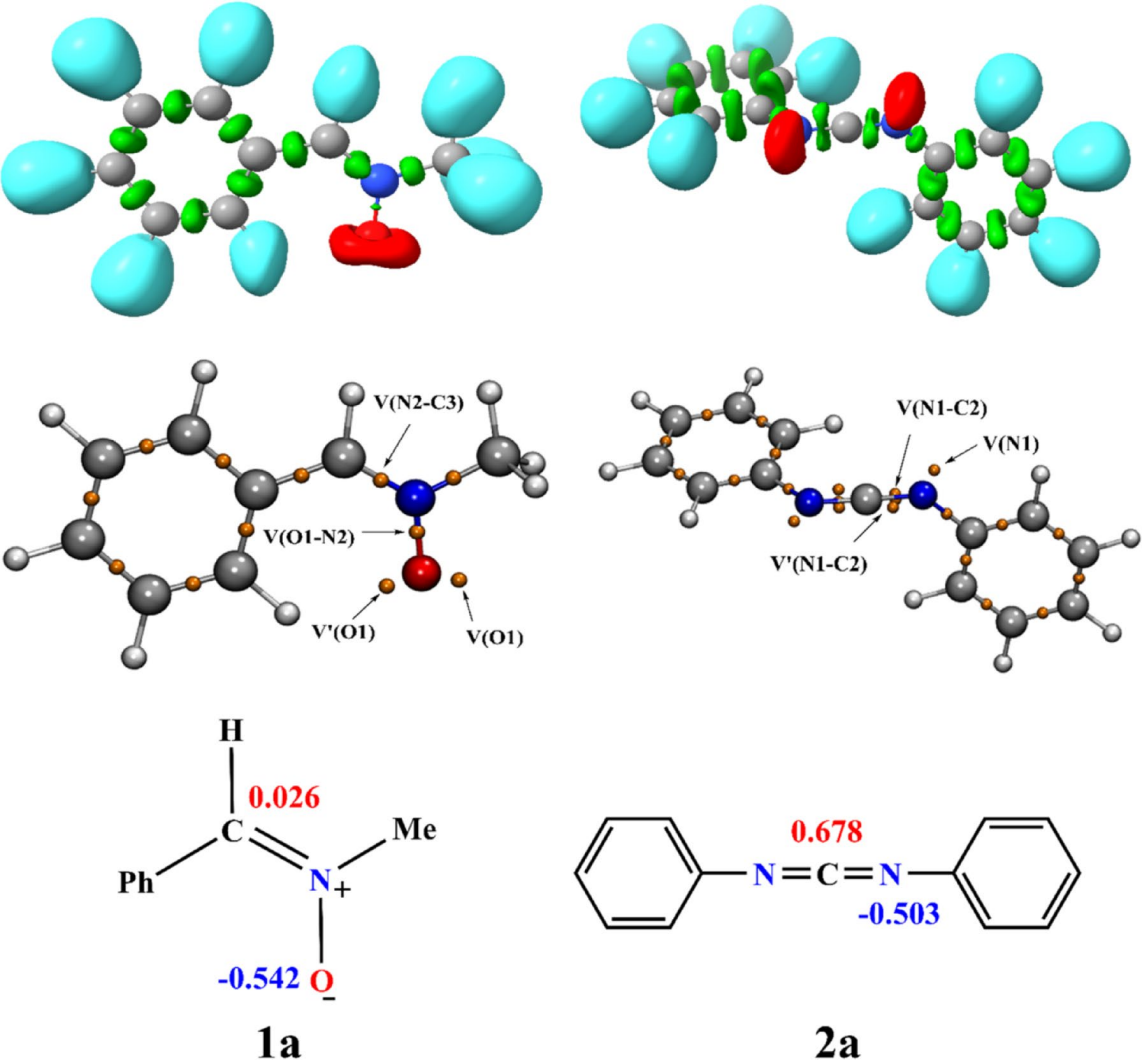
The Representation of ELF basins, attractor positions and atomic charges (NPA) in the Lewis structures of nitrone 1a and carbodiimide 2a.

The ELF isosurfaces and electron population distribution shown in Fig. 1 clearly demonstrate the regions of electron localization around the heteroatoms, particularly nitrogen and oxygen. The color scheme shows that blue represents the electronic distribution around hydrogen atoms, while red indicates non-bonding regions, especially localized free doublets on nitrogen and oxygen atoms. Table 1 gives the electron population values of the V(X) monosynaptic and V(X, Y) disynaptic basins for the nitrone (1a) and carbodiimide (2a) reagents. Topological analysis based on ELF values reveals the presence of two monosynaptic basins on oxygen V(O1) and V’(O1), totaling an electron population of 5.91 e. This relatively small value can be attributed to partial delocalization of the electron density, likely due to interaction with the adjacent aromatic π-system.

Furthermore, the presence of disynaptic basins between nitrogen N2 and carbon C3 (V = 4.05 e), as well as between oxygen O1 and nitrogen N2 (V = 1.31 e), confirms the formation of covalent bonds, a strong polarized bond O1-N2, and a very strong bond N2-C3, the latter most likely stabilized by conjugation with the aromatic nucleus. According to natural atomic charges, the nitrogen atom N2 carries a partial positive charge of + 0.026. In contrast, oxygen O1 carries a significant negative charge of −0.542, lending support to the partial zwitterionic character of nitrone 1a. The analysis with reagent 2a (carbodiimide) shows similar values for the N1 atom (V = 2.71 e-), and for the N1-C2 and C2-N3 bonds, with values of 1.83 e-, indicating a well-defined structure, but also one that is pre-organized for nucleophilic attack, typical of cycloaddition reactivity. Based on ELF values, natural charges and visualization of localization basins, reagent 1a can be classified as a type zwitterionic zw-type (or type III according to Domingo’s classification). Therefore, this ELF analysis allows us to precisely characterize the electronic properties of the reagent and predict its regioselectivity and stereoselectivity in 32CA reactions, including those with nitrones.

**Table 1:** Population valence basin values for nitrone 1a and carbodiimide 2a, in e.

V(O1)	1a	2a
	3.12	
V‘(O1)	2.79	
V(O1-N2)	1.31	
V(N2-C3)	4.05	
V(N1)	----	2.71
V(N1-C2)	----	1.83
V‘(N1-C2)		1.39
V(C2-N3)		1.83
V‘(C2-N3)		1.39
V(N3)		2.71

#### Analysis of the CDFT reactivity indices of the reagents

The theoretical approach conceptual of density functional theory (CDFT) makes it possible to quantitatively evaluate the chemical properties of molecules through global indices such as chemical hardness (η), chemical potential (µ), electrophilicity (ω) and nucleophilicity (N). These indices are crucial for evaluating a chemical species’ tendency to accept or donate electrons in a reaction. The classification of nucleophilic and electrophilic is based on the CDFT indices, and is classified according to the values of electrophilicity w and nucleophilicity N. Three intervals are crucial for classifying this species: nucleophilicity differentiates between strong electron donors (*N* > 3.00 eV), moderate electronophiles (2.00 < *N* < 3.00 eV), and marginal electronophiles (*N* < 2.00 eV). Classification according to strongly electrophilic (ω > 1.5 V), moderately electrophilic (0.8 < ω < 1.5 V), or weakly electrophilic (ω < 0.8 V).

**Fig. 2:**
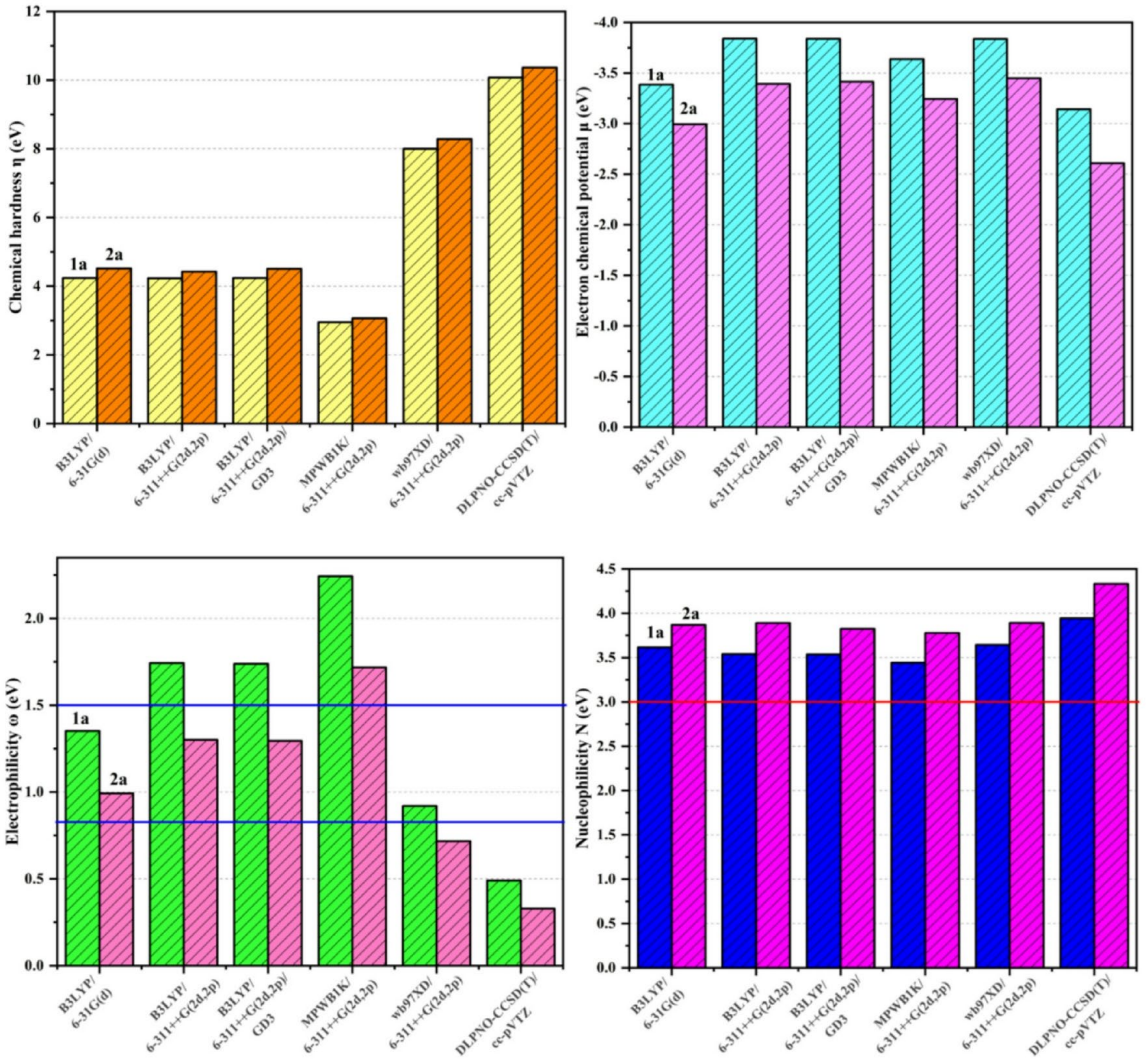
The CDFT indices (η, µ, ω, N) in eV of nitrone 1a and carbodiimide 2a reactants.

The reactivity analysis in Fig. 2 shows that CDFT provides the best presentation of the µ (eV) of reactants 1a and 2a in the cycloaddition reaction. A molecule’s tendency to accept or give up electrons is measured by its chemical potential (µ), which is more negative for 1a (−3.84 eV) than for 2a (−3.42 eV). The movement of electrons from 2a to 1a is suggested by the differential Δµ = µ(2a) - µ(1a) = 0.42 eV. In other words, nitrone 1a has the role of electron acceptor (electrophile) and carbodiimide 2a that of electron donor (nucleophile), indicating that this is a clearly defined polar reaction.

Chemical hardness (η), which estimates a molecule’s resistance to deformation of its electron density, is slightly greater for 2a (4.50 eV) than for 1a (4.24 eV). This indicates that 1a is more reactive than 2a from the perspective of electronic deformability, but the difference remains moderate. In contrast, it is the tendency to electron exchange, measured by µ, that dominates the polarity of the reaction here. With respect to the electrophilic index (ω), nitrone (1a) has a value of 1.74 eV, higher than that of carbodiimide 2a (1.30 eV), confirming the marked electrophilic character of nitrone 1a. We should remember that a molecule is considered a strong electrophile if ω(1a) > 1.5 eV. In addition, the nucleophilic index (N) is higher for 2a (3.83 eV) than for 1a (3.54 eV), confirming that 2a is a stronger nucleophile. This confirms that electrons were transferred from nucleophile carbodiimide 2a to electrophile nitrone 1a during the cycloaddition reaction.

In addition, as shown in Fig. 2, the B3LYP-D3/6–311 + + G(2d,2p) calculation approach used here proves highly computationally reliable, having analyzed the CDFT indices of reagents 1a and 2a. These methods employ a framework based on 2 d and 2p polarization and diffusion functions (++) and combine the hybrid functional B3LYP with a Grimme dispersion correction (D3), which is essential for an accurate description of non-covalent interactions. This results in fine modeling of overall reagent characteristics and electronic reactivity. The B3LYP-D3/6–311 + + G(2d,2p) method is an excellent option for the theoretical study of cycloaddition reactions because it establishes a balance between accuracy, computational cost and reliability compared with other more variable methods such as MPWB1K and wB97XD or more expensive ones such as DLPNO-CCSD(T).

**Fig. 3:**
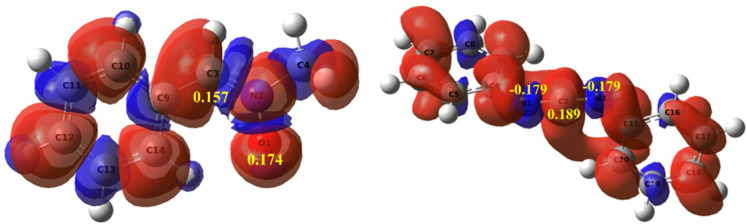
3D representations of the MASD of the radical anion $\:{2a}^{}$, and the radical cations $\:{1a}^{-}$ as well as the nucleophilic Parr functions $\:{P}_{k}^{-}\:$of the nitrone 1a and the electrophilic Parr functions $\:{P}_{k}^{+}\:$of carbodiimide 2a.

In 2013, Domingo and colleagues^49,50^ introduced the electrophilic and nucleophilic Parr functions, formulated on the basis of the Mulliken atomic spin density (MASD) assigned to the cation and anion radicals. It is well known that in cheletropic cycloaddition reactions involving unsymmetrical reactants, the preferred reaction pathway can be predicted by the nature of the most favorable two-center interaction between the nucleophilic and electrophilic reactants. In the case of [3 + 2] Cycloaddition Reactions Between Nitrone and Carbodiimide display in Fig. 3 the analysis of Parr indices based on local reactivity. These indices, derived from the frontier electron density, identify the molecular sites most susceptible to nucleophilic or electrophilic attack^51–53^. In molecule nitrone (1a) (electrophilic), $\:{P}_{O1}^{-}\:and\:\:{P}_{C3}^{-}\:$values indicate that atoms O1 (0.174) and C3 (0.157) are the most reactive centers, with O1 marginally more favorable to attack by a nucleophile. In comparison, reactant 2a shows that the C2 atom is a higher positive $\:{P}_{C2}^{+}$value of 0.189 marking this site as the main electron donor and the N1 and N2 nitrogen atoms show equal negative $\:{P}_{N1}^{+}={P}_{N2}^{+}=$−0.179 indicating a more electrophilic site in molecule 2a. This interpretation indicates that the regioselectivity of the 32CA reaction between nitrone and carbodiimide aligns with experimental results, specifically showing that the C2 and O1 atoms of nitrone 1a are consistent with these findings.

#### Mechanistic pathways in the cheletropic cycloaddition reactions of nitrone 1a and carbodiimide 2a

**Scheme 2:**
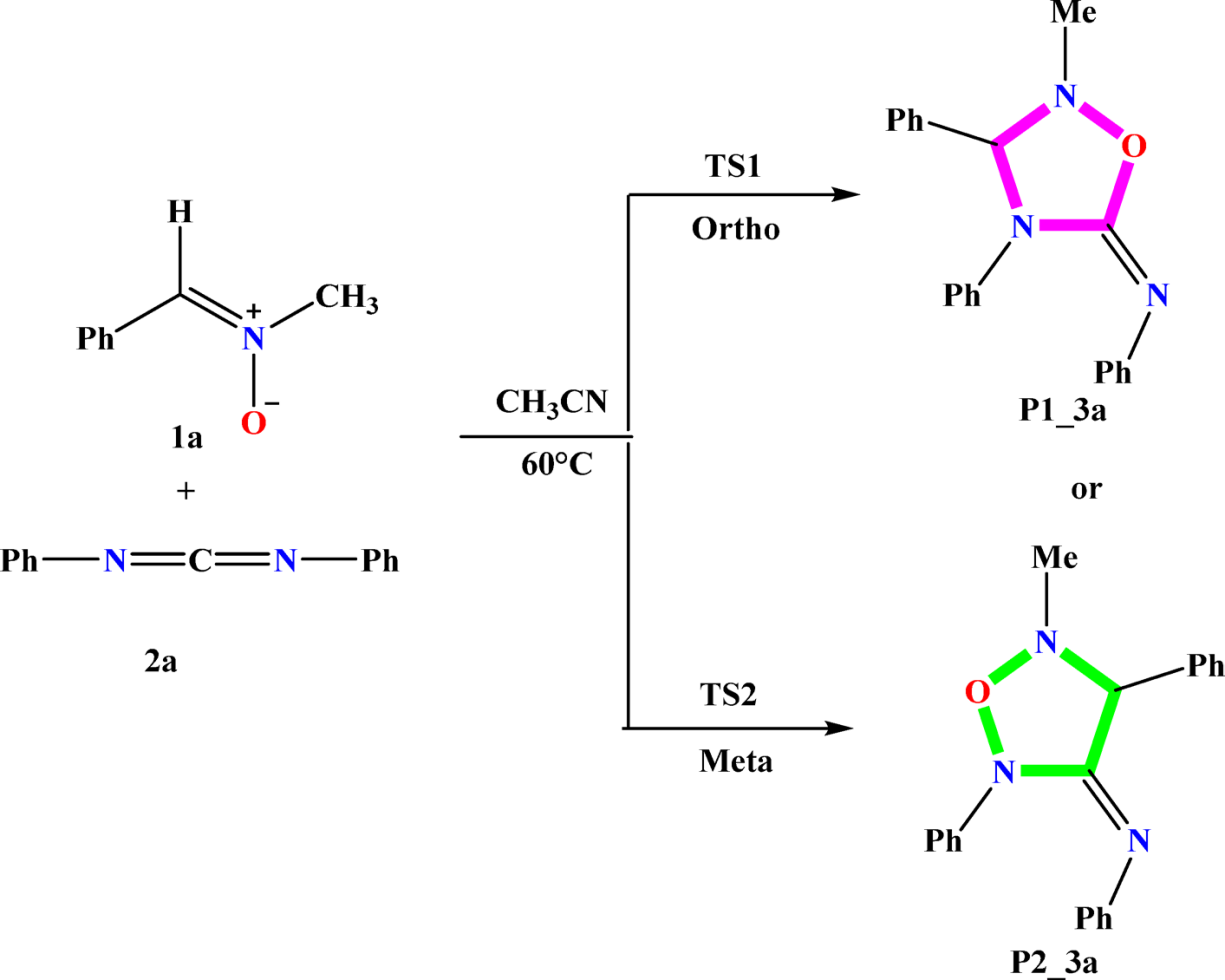
32CA reactions of carbodiimide 2a and nitrone 1a studied.

A thorough analysis of ELF results and CDFT indices provides a detailed understanding of the reactivity and polarity of the 32CA between nitrone 1a and the asymmetric dipolarophile, carbodiimide (2a). This reaction, which also proceeds via two distinct transition states (TS1 and TS2) at 60 °C, could in theory be carried through to two cycloadduct regio-isomers P1_3a (ortho) and P2_3a (meta). The asymmetrical nature of the dipolarophile 2a enables a marked regioselectivity. Reactivities allow us to predict the preferred direction of attack. The O1 oxygen atom of the nitrone exhibits a high localized electron density, marking an active nucleophilic site, while the C2 carbon of 2a, identified as the electrophilic center, is susceptible to attack. This favorable interaction, enhanced by π-conjugation, preferentially leads to the formation of the P1_3a product (ortho regioisomer) via the TS1 transition state, as shown in Scheme 2.

**Fig. 4:**
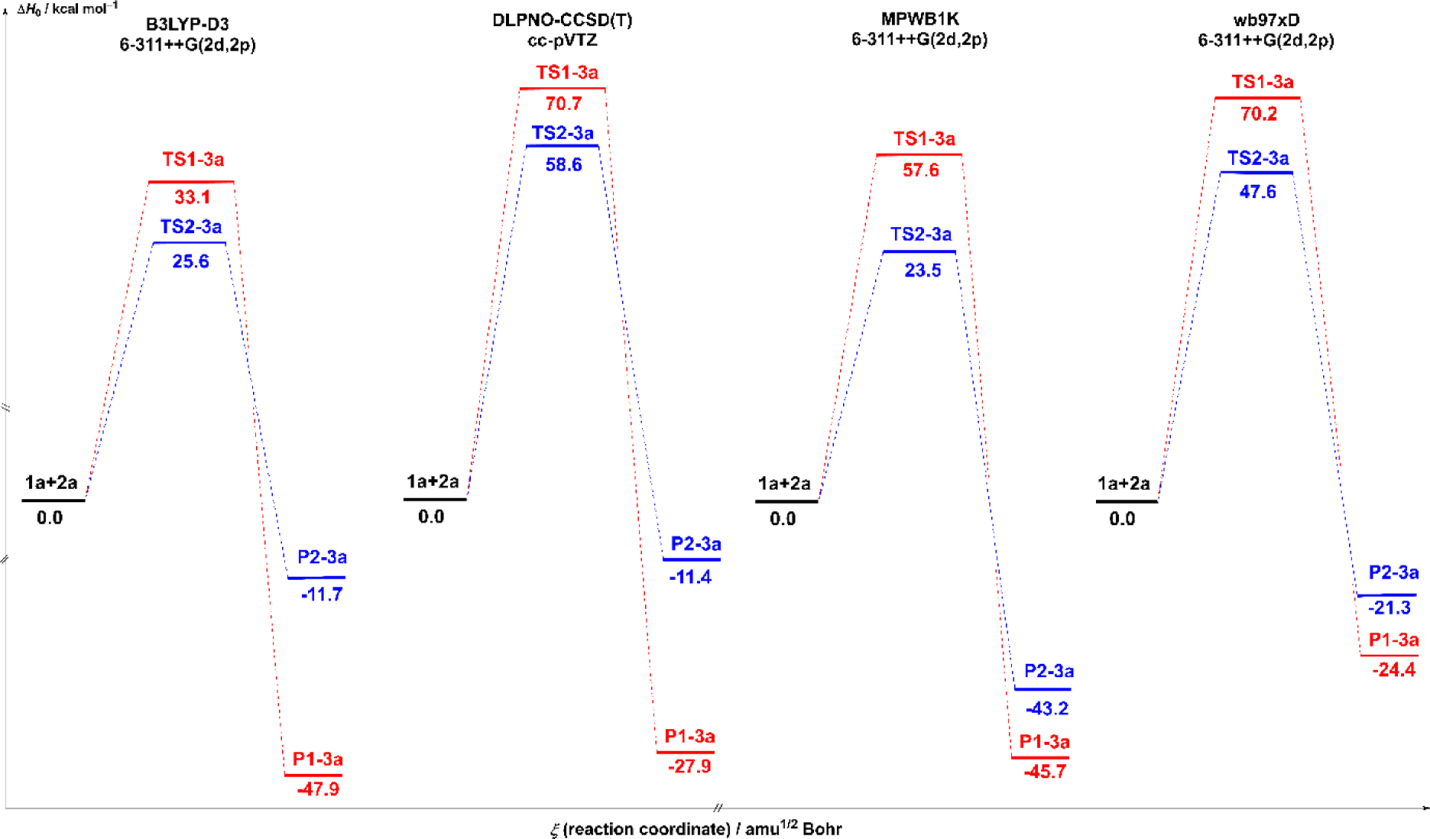
The energy profiles in gas-phase for the transition states and products of the reaction between nitrone 1a and carbodiimide 2a, calculated at the different theoretical levels B3LYP-D3, MPWB1K, WB97XD with site basis 6–311 + + G(2d,2p) and DLPNO-CCSD(T). The corresponding values are given in Table 1 and S2 of the supplementary document.

**Fig. 5:**
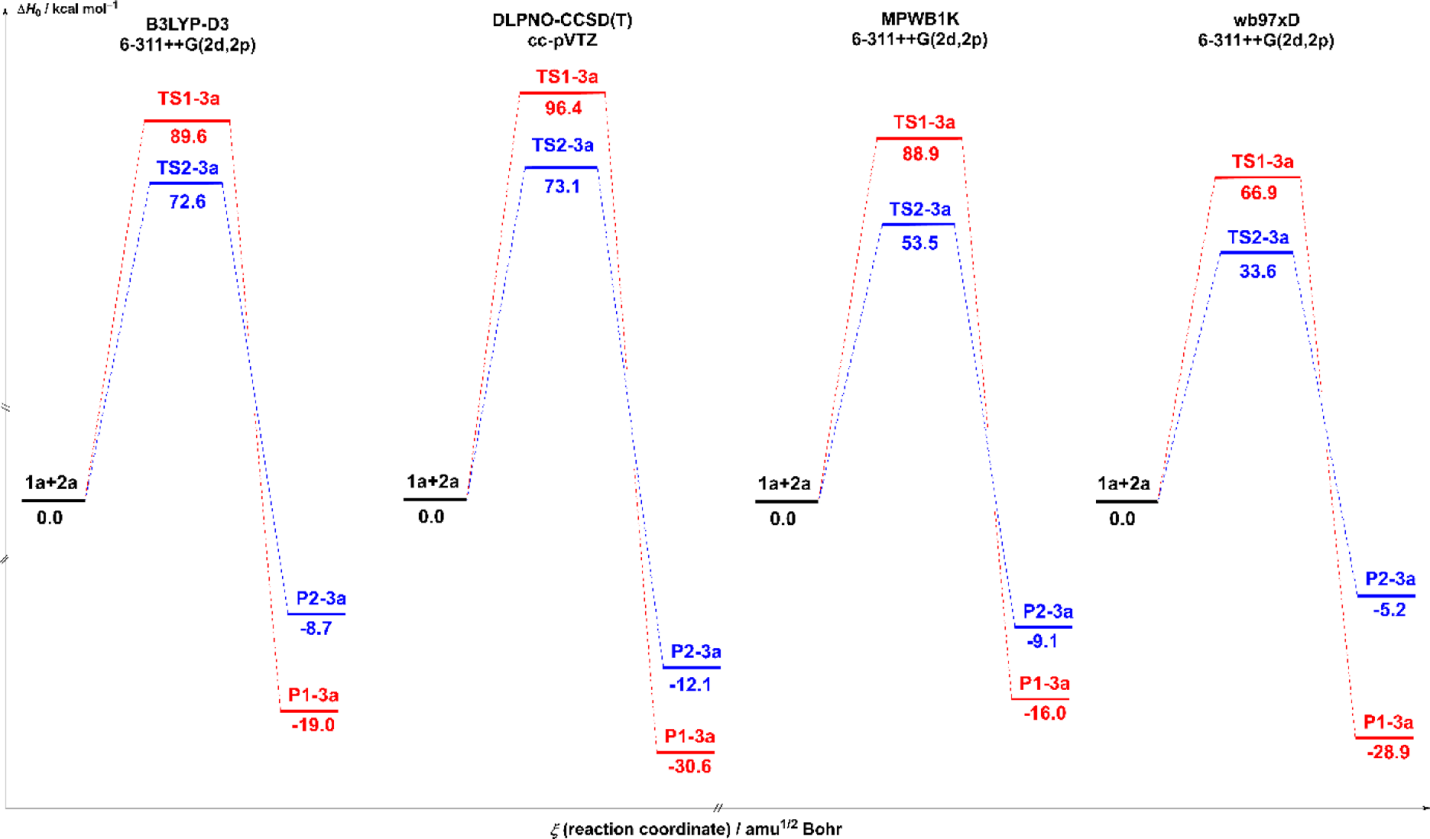
The energy profiles in solvent acetonitrile for the transition states and products of the reaction between nitrone 1a and carbodiimide 2a, calculated at the different theoretical levels B3LYP-D3, MPWB1K, WB97XD with site basis 6–311 + + G(2d,2p) and DLPNO-CCSD(T). The corresponding values are given in Tables S1 and S2 of the supplementary document.

The enthalpy energy profiles (ΔH, in kcal/mol), shown in Figs. 4 and 5, provide a means of analyzing the evolution of energy along the reaction path of the transformation of 1a + 2a to two potential products, P1-3a and P2-3a, via two distinct transition states: TS1-3a and TS2-3a. These profiles were studied at a temperature of 333 K and a pressure of 1 atm, in the gas phase (Fig. 1) and in solution in acetonitrile (Fig. 2), using different calculation methods: B3LYP-D3, DLPNO-CCSD(T), MPWB1K and wB97XD with the base 6–311 + + G(2d,2p) or cc-pVTZ, depending on the method.

In the gas phase, the activation barriers (ΔH) associated with TS1-3a transition states are significantly higher than those for TS2-3a for all the methods studied. For example, the B3LYP-D3 method predicts a barrier of 89.6 kcal/mol for TS1-3a versus 72.6 kcal/mol for TS2-3a, as shown in Table 2. This trend is confirmed by DLPNO-CCSD(T) (96.4 vs. 73.1), MPWB1K (88.9 vs. 53.5), and wB97XD (66.9 vs. 33.6). This suggests that the pathway leading to product P2-3a is kinetically more accessible in the gas phase. However, these kinetic findings must be complemented by athermodynamic analysis of the products. The study of product enthalpies shows that the product P1-3a is more stable than P2-3a in all conditions, with ΔH ranging from − 16.0 to −30.6 kcal/mol for P1-3a, versus − 5.2 to −12.1 kcal/mol for P2-3a. So, even though the path to P2-3a is kinetically easier, P1-3a is still the thermodynamically favored product. The ΔG values for P1-3a and P2-3a are both negative, indicating that product formation is thermodynamically favorable. However, P1-3a still has a more negative ΔG value than P2-3a, confirming its greater stability. Similarly, enthalpy values (ΔH) are lower for P1-3a, reinforcing this conclusion.

As shown in the energy profiles in Fig. 4, the activation barriers associated with TS2-3a are consistently lower than those of TS1-3a, indicating that the formation of P2-3a is kinetically favored. In contrast, the energies of the end products reveal that P1-3a is thermodynamically more stable than P2-3a, with more negative ΔH and ΔG values regardless of the calculation metho, as shown in Table 2 and Tables S1 and **S2**. Thus, the reaction is governed by kinetic control in favor of P2-3a, while P1-3a corresponds to the product under thermodynamic control, clarifying the distinction between kinetic accessibility and product stability.

The polar aprotic solvent acetonitrile significantly modifies the energy profiles, with a general decrease in ΔH and ΔG for products and transition states, showing that the solvent stabilizes the entire reaction profile. With the B3LYP-D3 method, the activation barriers drop drastically: TS1-3a decreases from 89.6 to 33.1 kcal/mol, and TS2-3a decreases from 72.6 to 25.6 kcal/mol. This significant drop indicates that the solvent effectively stabilizes the transition states, thereby facilitating the progress of the reaction. Similar behavior is observed with MPWB1K, where the barriers decrease sharply, particularly TS2-3a (from 53.5 to 23.5 kcal/mol). This shows that the solvent reduces the energetic constraints on the reaction, particularly in DFT-based functional methods, which is consistent with the known effects of polar solvents on reactions involving polar or charged transition states. Another improvement is the stability of the product in solvent, namely for P1-3a. The enthalpy of P1, for example, increases from − 19.0 kcal/mol (gas phase) to −47.9 kcal/mol (acetonitrile) with B3LYP-D3, signaling considerable stabilization. The same applies to P2-3a, but to a lesser and more limited extent. In the case of particular methods, such as MPWB1K, the difference in stability between P1-3a and P2-3a becomes extremely small in solution terms (−45.7 vs. −43.2 kcal/mol), suggesting possible competition between the two products. However, P1-3a remains the overall thermodynamically favored product, even in the presence of the solvent.

To ensure a more rigorous kinetic analysis, the Gibbs free energies of activation (ΔG) were evaluated systematically for all the reaction pathways studied. The activation barriers in the gas phase are 43.82 kcal/mol for TS1-3a and 20.48 kcal/mol for TS2-3a, as shown in Table 2, indicating that the pathway leading to product P2-3a is kinetically favored. In aqueous acetonitrile solution, these barriers decrease significantly to 20.48 kcal/mol and 9.74 kcal/mol, respectively, indicating the stabilizing effect of the solvent on the transition states. The ΔG values are therefore more important than the ΔH values. Thus, although the enthalpy profiles shown in Figs. 4 and 5 allow for a rough comparison between the different theoretical methods, the kinetic conclusions are based on the Gibbs free energy barriers listed in the tables Table 2 and Tables S1-S2.

**Table 2:** Thermodynamic properties of transition states (TS) and products (P) of 32CA reactions of carbodiimide 2a and nitrone 1a in gas phase at 333 K and 1 atm, relative energies (ΔE), enthalpies (ΔH), and Gibbs free energies (ΔG) in kcal.mol^− 1^ and ΔS) in cal.mol^− 1^.K^− 1^.

TS/*P*	Phase	∆E	∆H	∆G	∆S	GEDT
TS1-3a	Gas phase	89.64	21.78	43.82	−66.16	0.08
P1-3a	Gas phase	−18.98	−79.48	−62.31	−51.52	
TS2-3a	Gas phase	72.60	6.67	24.18	−52.56	0.09
P2-3a	Gas phase	−8.66	−45.22	−28.04	−51.58	
TS1-3a	Acetonitrile	33.13	28.83	20.48	−64.52	0.14
P1-3a	Acetonitrile	−47.91	−45.67	−30.05	−46.88	
TS2-3a	Acetonitrile	25.63	23.55	9.74	−48.15	0.15
P2-3a	Acetonitrile	−11.67	−9.86	−24.76	−44.87	

The Global Electron Density Transfer (GEDT) measures the amount of electrons transferred between two reactants in the transition state (see Table 2 and Tables S1-S2). Values below 0.05 e indicate a non-polar mechanism, between 0.05 e and 0.2 e a low-polar mechanism, and above 0.2 a high-polar mechanism. In your results, GEDT varies from 0.08 e to 0.15 e, indicating that the reaction is strongly polar. An observed increase in GEDT occurs in solution (acetonitrile), indicating that the solvent enhances the polarization of the transition state. Electron flow is from nitrone 1a to reagent 2a, confirming a direct polar mechanism favored in polar media, consistent with CDFT results.

#### Bonding evolution theory (BET) at the formation P1_3a

The evolution of the reaction based on intrinsic reaction coordination (IRC) starts with the MC-I (complex) structure, which is defined by long distances between the atoms of reactants 1a and 2a (d(O1-C5) = 3.09 Å and d(C3-N4) = 3.13 Å), suggesting the absence of bonding. These values are mentioned in Table S3, and the detailed reaction mechanism is shown in Scheme S1. During stages I to III, these distances beginning to decrease noticeably (down to 1.59 Å for O1-C5 and 2.35 Å for C3-N4 in stage S3 i.e. Phase III), and also notice the presence of monosynaptic basin in the nitrogen atom N2 at a population value equal to 1. 39e, accompanied by a considerable increase in GEDT (up to 0.43 e), representing a significant charge transfer for the interaction between 1,3-dipole nitrone 1a and carbodiimide 2a. After Phase III comes Phase VI, whose main feature is the increasing presence of the monosynaptic basin carbon V(C5), which in turn contributes to the single bond with the oxygen O1 (of nitrone 1a) and this bond is formed in Phase V as shown by the presence of the disynaptic basin between this C5 atom and O1 at a value of 1.10 e, at a GEDT equal to 0.43 e. In the final phases VII and VIII (S7 and S8), the critical distances O1-C5 and C3-N4 approach the values of standardized monobonds (1.41 Å and 1.53 Å respectively), while the values of V(O1,C5) and V(C3,N4) increase, validating the creation of new bonds. The product P1-3a finally has optimized distances (d(O1-C5) = 1.35 Å and d(C3-N4) = 1.29 Å) and a relative energy ΔE = −18.98 kcal/mol, indicating a stable, exergonic structure, which confirms the cycloaddition undertaken with the proposed mechanism.

#### QTAIM topological analysis at TSs

Following the BET study, the Non-Covalent Interactions (NCI) analysis enables the provision of additional clarity regarding the remarkable selectivity observed in the 32CA. The NCI approach is dependent on the conceptualization of low Reduced Density Gradient (RDG) equidensity surfaces associated with the standard product denoting the second value of the electron density, ρ(ƛ₂)^54^. This method enables the characterization of various failed reactions within the reactivity space^55,56^.

**Fig. 6:**
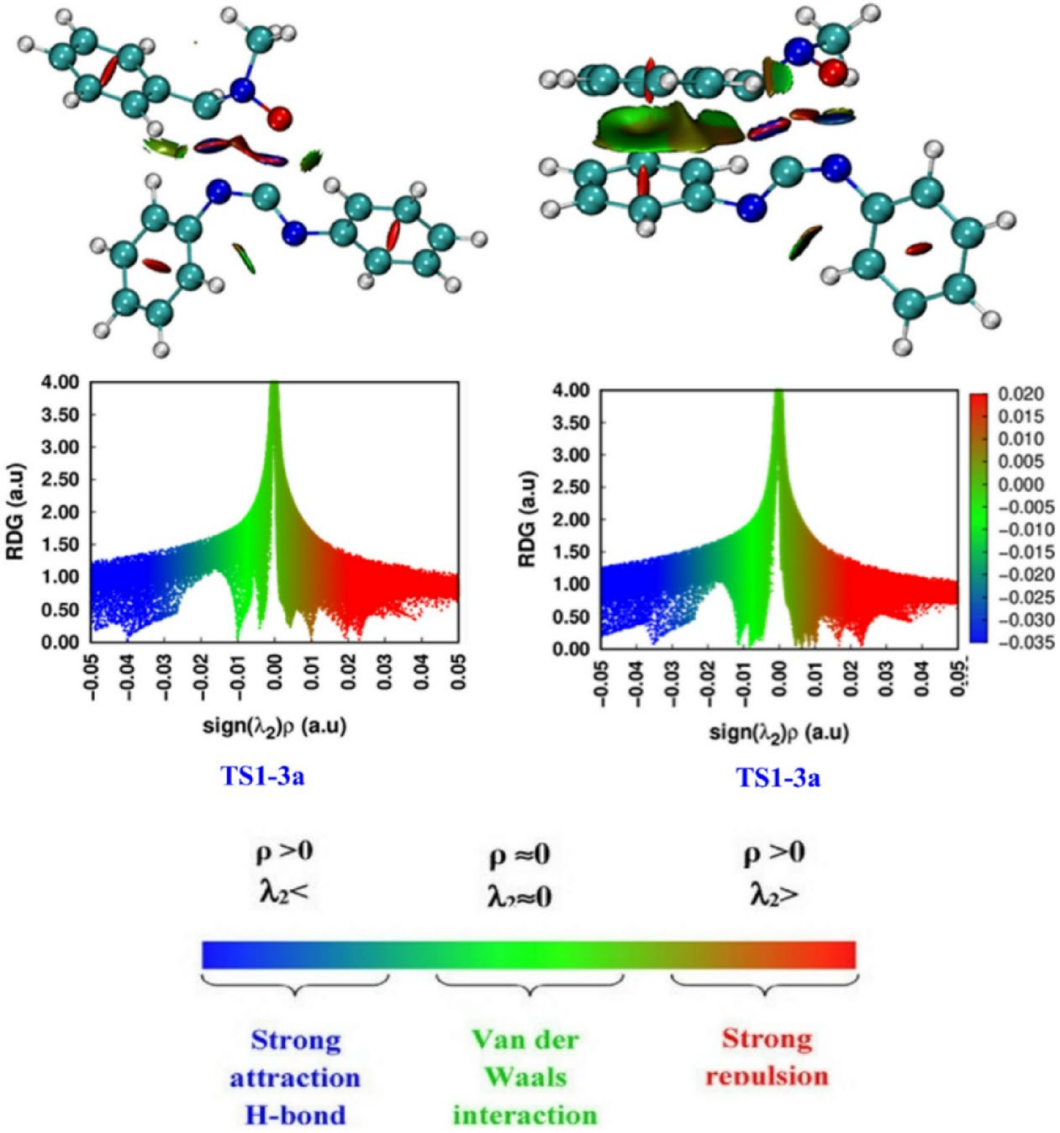
Non-Covalent Interactions and RDG of the TSs associated with the 32CA reaction of nitrone (1a) with carbodiimide (2a), (isovalue = 0.20).

Analysis of the NCI-enabled transitions detects clear differences in the two possible orientations of the 1,3-dipolar cycloadduct between nitrone 1a and carbodiimide 2a. The isodensity surfaces and RDG plots denote the stabilization of the transition state to the product P1-3a through appealing non-covalent interactions, namely hydrogen bonds and Van der Waals forces (blue areas and vertices) as presented in Fig. 6. In contrast, the alternative state exists in regions with repulsive steric forces (red zones), due to the favorable affinity between bulky groups such as the phenyl and methyl groups. This repulsion results in increasing the energy of the transition state and also renders it more favorable. The study of the effect of genetic influences verifies that the preferred transition state is one in which the involved groups are spatially well-accessible, irrespective of electronic barriers. In the favorable TS1-3a case, the substitutions are positioned to minimize absolute strict oppositions while optimizing stable interactions. This configuration enables efficient geometric modification between the two reactions, reducing the reaction barrier. However, the experimentally observed selectivity is explained by the non-covalent nature of the responses and by the spatial distribution of the radicals, with this stable transition state still more probable to lead to the final product.

### Prediction of the products 1,2,4-oxadiazolidine from the 32CA reaction between nitrone and carbodiimide (2a)

#### Quantum ESP and ELF calculations for the compound

**Fig. 7:**
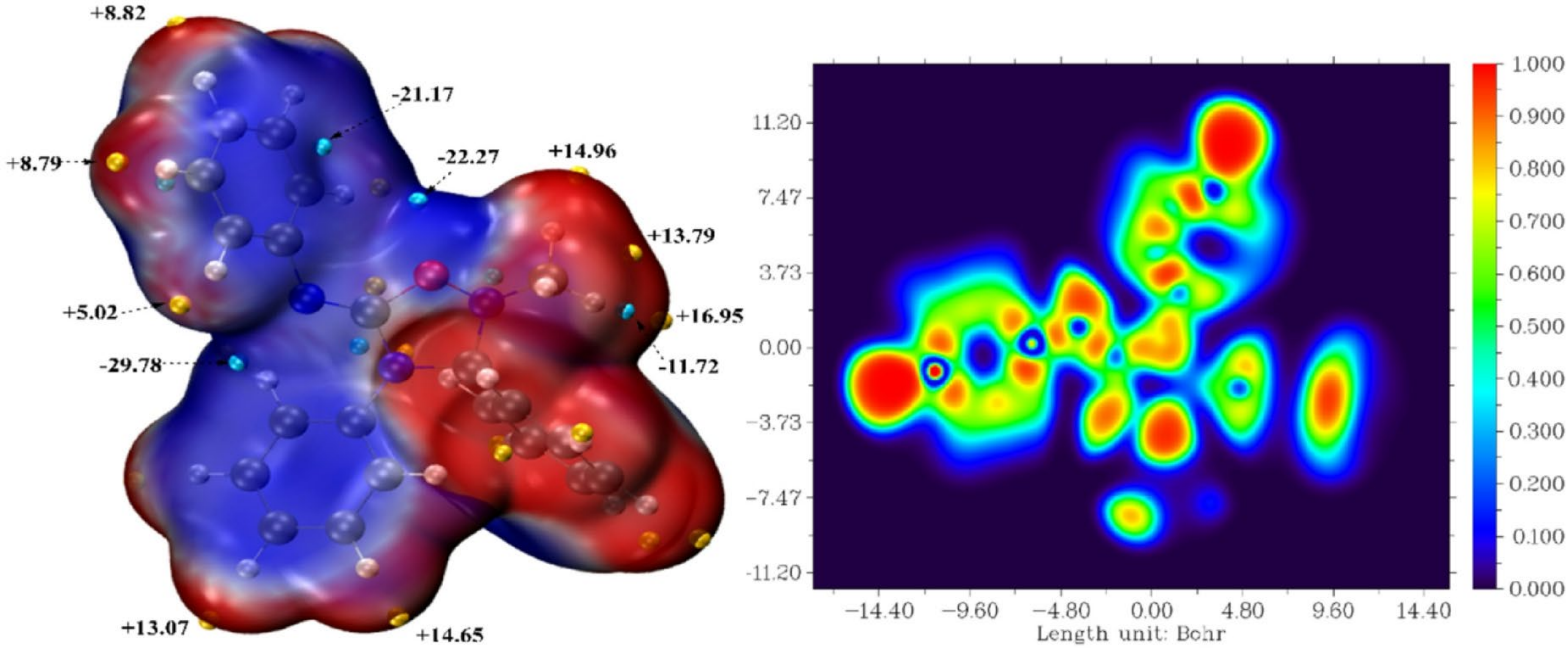
ESP-mapped and ELF map of cycloadduct 1,2,4-oxadiazolidine.

In silico analysis of the compound 1,2,4-oxadiazolidine (P1_3a) was conducted using two approaches: electronic localization function (ELF) and electrostatic surface potential (ESP). (see Fig. 7). The combined analysis of the ELF and ESP of the studied candidate compound has provided essential information on its chemical reactivity and its capability to interact selectively targeted biological products^57,58^. The ELF map reveals several areas with a high concentration of electrons (ELF values between 0.9 and 1.0), indicating the presence of stable covalent bonds and non-bonding doublets, primarily around heteroatoms such as oxygen and nitrogen atoms. At another level, ESP mapping reveals a polarized distribution of electrostatic potential at the molecule’s surface, highly nucleophilic (red zones) to, electrophilic (blue zones). This electrostatic heterogeneity favors the establishment of specific directional interactions with the protein’s active site residues, such as hydrogen bonds, ionic interactions and van der Waals forces. Thus, highly localized ELF regions coinciding with ESP zones of high negative potential indicate optimal reactive sites for the formation of stable complexes with basic protein residues. In contrast, zones of high positive potential can interact with acidic residues. The compound is a promising candidate for the development of new treatments, particularly in terms of therapeutic selectivity and affinity, as demonstrated by its strong interaction with the target protein. The addition of these two approaches to our research enables us to identify precisely the most suitable targets for molecular drug interaction, helping to facilitate the design of effective ligands.

#### Molecular docking

**Fig. 8:**
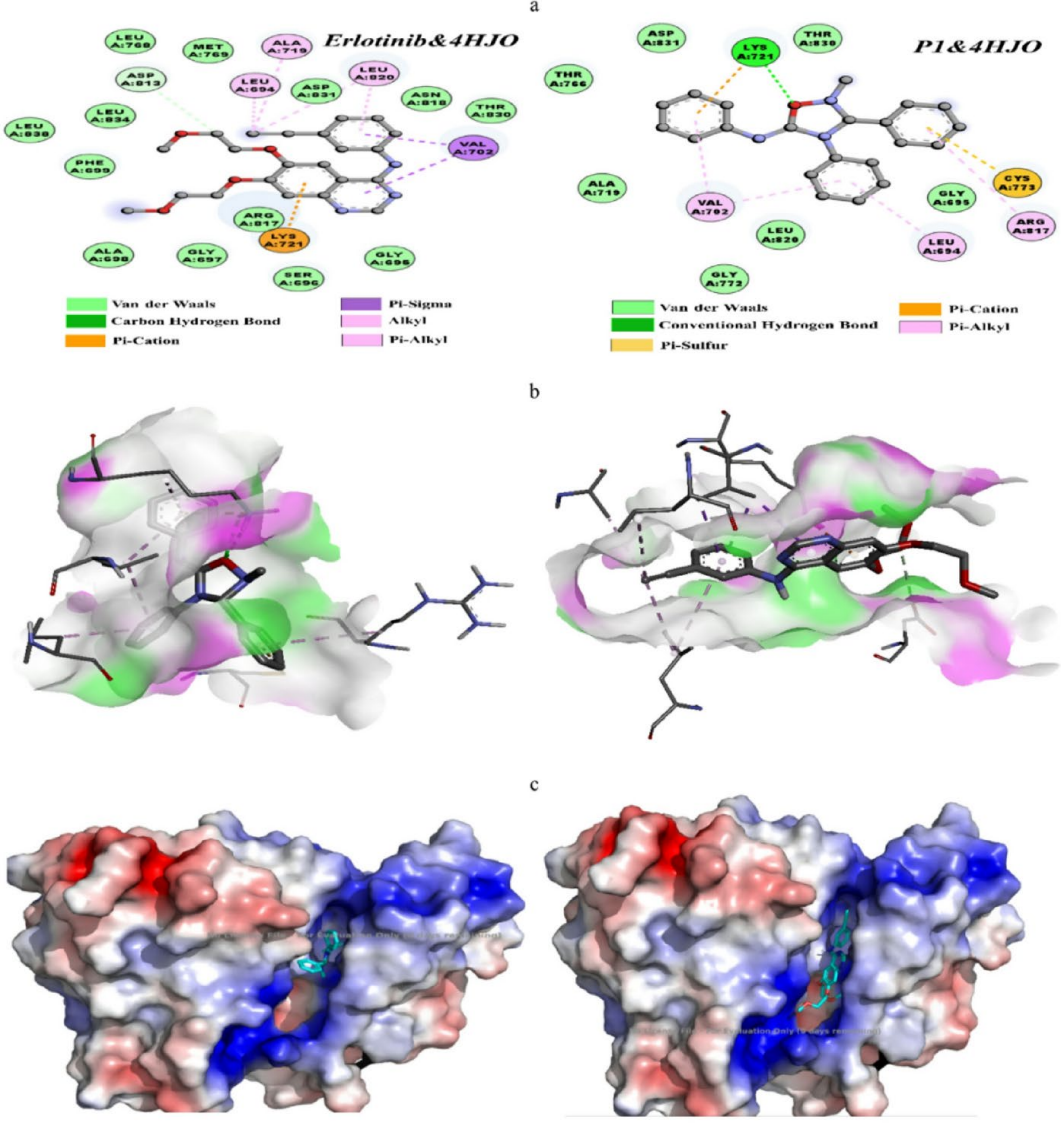
Structural and electrostatic analysis of the designed complex: (**a**) Two-dimensional (2D) Representation, (**b**) Three-dimensional (3D) Visualization, and (**c**) Electrostatic potential map (APBS) for the interaction of compound P1 and Erlotinib with EGFR (PDB: 4HJO).

After examining the electronic reactivity of the compound using electrostatic potential surface (ESP) and electronic localization function (ELF), the molecular docking was performed to appreciate its interaction with the EGFR passive domain promoter domain (PDB:4HJO), with Erlotinib as a co-crystallized compound ligand. The electrostatic and nucleophilic regions of the ligand were identified using ESP mapping, which guided its optimal orientation in the activated site. ELF analysis identified areas of particularly high electron density, which are favorable for non-covalent interactions with specific target protein residues, particularly through hydrogen bonds or π interactions. In Fig. 8, we present the docking results for: (a) docking positions, (b) surface hydrogen bonding, and (c) electrostatic APBS of the designed complex, including the designed compound (P1) and Erlotinib 4HJO. The compound P1 studied exhibits a good affinity for the EGFR active protein, with a binding energy of −8.8 kcal/mol, which is more stable than that of Erlotinib (−7.0 kcal/mol), with a difference of approximately 1.8 kcal/mol. The molecular docking results reveal several essential interactions between the P1 ligand and the 4HJO target protein, including hydrogen bonding with the LYS721 amino acid, as well as a hydrophobic interaction involving a p-cation and p-sulfur interaction with CYS773, suggesting a novel and potentially more specific binding mode. On the other hand, co-crystallized (Erlotinib) shows the absence of hydrogen bonding, but the presence of several interactions with the amino acids VAL702, LU694, and LU820, LYS 721, ALA 719 of the Pi-alkyl Pi-Sigma, Pi-cation, and alkyl types. Both ligands display numerous van der Waals interactions. However, the product P1 illustrates a diversity of interactions that could suggest an inhibitory ability comparable or even in relation to that of Erlotinib, depending on the results of in vivo activity, which require confirmation.

The results of the H-Bond surface analysis for both complexes clearly illustrate the interaction regions through hydrogen bonds, highlighting the acceptor zones in green and the donor zones in pink. Finally, an electrostatic examination of the protein surface using the APBS calculation demonstrated that the ligand tends to locate itself in an electropositive cavity (shown in blue), which is conducive to interaction with its electronegative zones, thereby validating adequate electrostatic complementarity between the ligand and the target. These observations suggest a strong affinity of the ligand for the catalytically inactive domain of EGFR, aligning with the known mechanism of action of tyrosine kinase inhibitors like Erlotinib.

#### ADMET analysis

The properties of absorption, distribution, metabolism, excretion, and toxicity (ADMET) are crucial parameters in the preclinical assessment of drug candidates, as shown in Table 3. They help predict how a molecule will be absorbed, distributed throughout the body, metabolized, and excreted, as well as whether it may exhibit toxic effects. The two studied molecules analyzed, Erlotinib (a known drug) and P1_3a (a new compound), displayed excellent oral absorption, with high cellular permeability and intestinal absorption of more than 90%. Both are glycoprotein-P substrates, which may limit their efficacy by promoting their elimination from cells. P1_3a shows better distribution in the CNS (better blood-brain barrier crossing), which may represent an advantage for some therapeutic indications). Regarding metabolism, both molecules are broken down by the CYP3A4 enzyme, but only Erlotinib also acts as an inhibitor, increasing the risk of drug interactions. In terms of excretion, Erlotinib has a significantly higher clearance rate, leading to faster elimination rate. In terms of toxicity, both molecules are non-mutagenic (negative AMES test result) and non-toxic to the liver and heart (do not inhibit hERG). That being said, P1_3a is a little better tolerated, with a higher maximum dose and lower environmental toxicity. In conclusion, although both compounds are safe and promising, P1_3a an overall comparable ADMET profile, making it a more interesting candidate for future development.

**Table 3:** ADMET parameters of designed compound P1 and Erlotinib.

Compounds	Caco2 permeability (10⁻⁶ cm·s⁻¹)	Intestinal absorption (%)	*P*-glycoprotein substrate	*P*-glycoprotein inhibitor	BBB	CYP2D6 substrate	CYP3A4 substrate	CYP2D6 inhibitior
Erlotinib	1.064	90.588	Yes	Yes	−0.427	No	Yes	No
P1_3a	1.08	93.61	Yes	Yes	0.272	No	Yes	No
Compounds	Total clearance (mL·min⁻¹·kg⁻¹)	Renal OCT2 substrate	AMES toxicity	Max. tolerated dose (mg·kg⁻¹·jour⁻¹)	hERG I inhibitor	Hepatotoxicity	*T.Pyriformis* toxicity (µg·L⁻¹)	Minnow toxicity (mmol·L⁻¹)
Erlotinib	0.628	No	No	0.306	No	No	0.707	0.547
P1_3a	0.127	No	No	0.387	No	No	0.766	0.534

#### Molecular dynamics simulations

To further validate the stability of the selected protein-ligand complex beyond the static molecular docking results, a 100 ns molecular dynamics (MD) simulation was performed. This approach provides a dynamic perspective on the behavior of the complex under near-physiological conditions, allowing the assessment of conformational stability, structural compactness, residue flexibility, and interaction persistence over time. In this study, the stability of the complex was evaluated using several key parameters, including root mean square deviation (RMSD), root mean square fluctuation (RMSF), hydrogen bond analysis, radius of gyration (Rg), solvent-accessible surface area (SASA), and total energy.

**Fig. 9:**
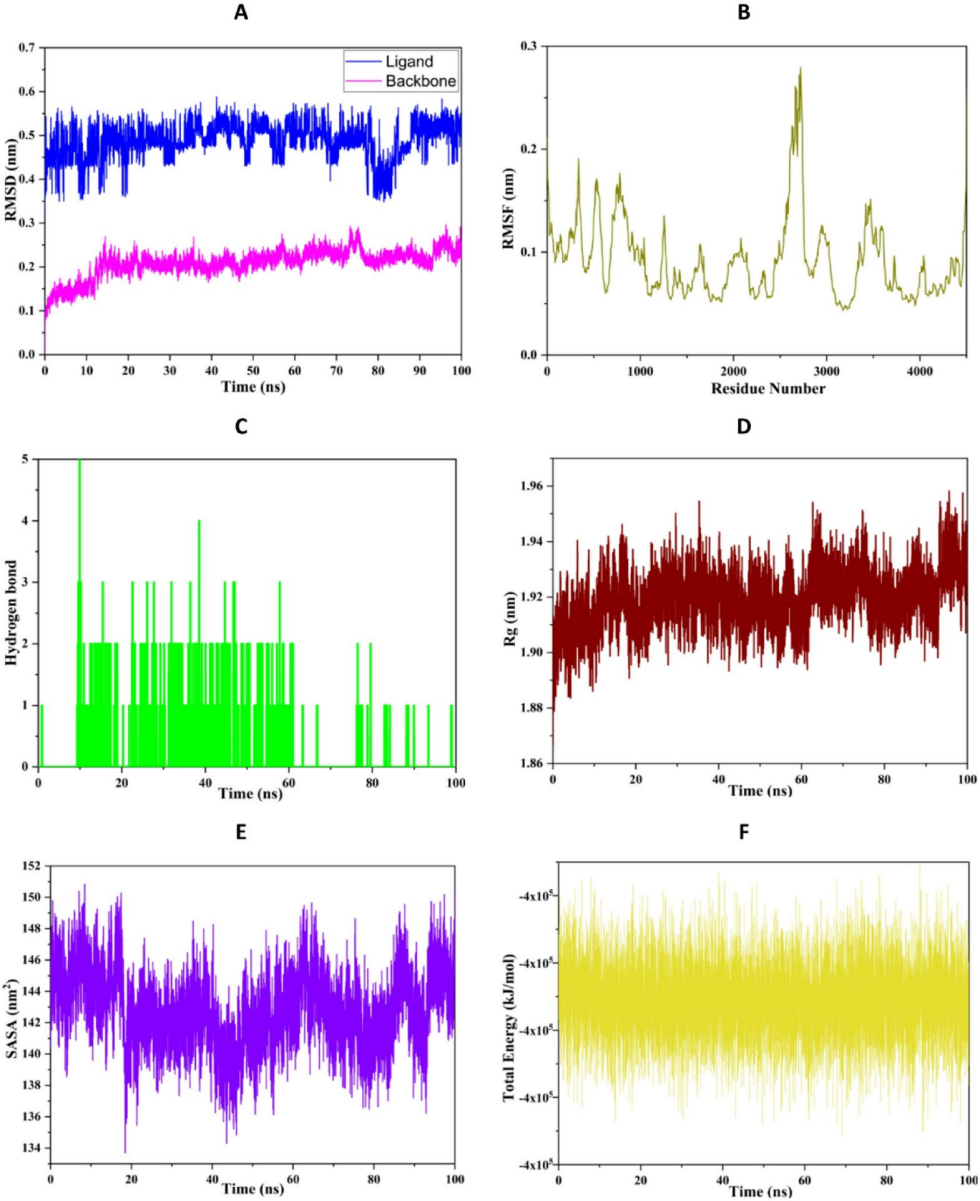
Molecular dynamics simulation of representative ligand and protein complexes calculated from a 100 ns simulation.

##### RMSD analysis

The root mean square deviation (RMSD) was calculated to evaluate the structural stability and conformational behavior of both the protein backbone and the bound ligand throughout the 100 ns molecular dynamics simulation (Fig. 9A). The backbone RMSD exhibits an initial equilibration phase within the first ~ 10 ns, during which the system rapidly adjusts from its starting conformation, reaching values around 0.18–0.20 nm. Following this phase, the RMSD stabilizes and fluctuates within a narrow range of approximately 0.20–0.25 nm for the remainder of the simulation. This limited deviation indicates that the protein maintains a stable global conformation, with no significant structural unfolding or instability observed over time. The small amplitude of fluctuations further suggests that the system has reached equilibrium and remains well-converged. In contrast, the ligand RMSD shows slightly higher values, fluctuating between ~ 0.40 and 0.55 nm throughout the simulation. These variations reflect the inherent flexibility of the ligand within the binding pocket, including minor positional adjustments and conformational reorientations. Notably, despite these fluctuations, the ligand RMSD does not exhibit any abrupt increases or continuous drift, indicating that the ligand remains consistently bound within the active site without dissociation. A transient decrease in ligand RMSD observed around 75–85 ns suggests a temporary stabilization or tighter binding conformation, possibly due to optimized intermolecular interactions such as hydrogen bonding or hydrophobic contacts. This behavior further supports the dynamic yet stable nature of the ligand-protein interaction.

##### RMSF analysis

The root mean square fluctuation (RMSF) was calculated to evaluate the residue-level flexibility and dynamic behavior of the protein during the 100 ns molecular dynamics simulation (Fig. 9B). Overall, the RMSF profile reveals that the majority of residues exhibit relatively low fluctuations, predominantly within the range of ~ 0.05 to 0.12 nm. This indicates that the protein maintains a high degree of structural rigidity throughout the simulation, particularly within the core regions that are typically associated with secondary structural elements such as α-helices and β-sheets. Several distinct peaks are observed in the RMSF profile, reflecting localized regions of increased flexibility. Notably, higher fluctuations reaching up to ~ 0.25–0.28 nm are detected around specific residue segments (e.g., approximately 2600–2700), suggesting the presence of highly dynamic loop regions or solvent-exposed domains. These flexible regions are commonly associated with functional motions, including ligand accommodation, allosteric regulation, or conformational adaptability. In contrast, residues corresponding to the binding pocket exhibit comparatively lower RMSF values, indicating restricted mobility and structural stabilization upon ligand binding. This reduced flexibility in the active site region supports the formation of stable protein-ligand interactions, consistent with the RMSD results and further confirming the integrity of the complex during the simulation. Additionally, moderate fluctuations observed at the N- and C-terminal regions are expected due to their intrinsic flexibility and exposure to the solvent environment. Importantly, no excessive or abnormal fluctuations were detected, suggesting the absence of structural instability or unfolding events.

##### Radius of gyration

The radius of gyration (Rg) was calculated to assess the overall compactness and structural integrity of the protein throughout the 100 ns molecular dynamics simulation (Fig. 9C). The Rg profile remains relatively stable over the simulation time, fluctuating within a narrow range of approximately ~ 1.90 to 1.95 nm. This limited variation indicates that the protein maintains a consistent level of compactness without undergoing significant expansion or contraction. The absence of large deviations or abrupt transitions suggests that no major conformational rearrangements or unfolding events occur during the simulation. A slight increase in Rg values observed toward the later stages of the trajectory (after ~ 70 ns) may reflect minor structural adjustments or breathing motions, which are commonly associated with protein flexibility in solution. However, these fluctuations remain within an acceptable range and do not compromise the overall structural stability.

##### H-bond analysis

Hydrogen bond analysis was performed to evaluate the stability and persistence of intermolecular interactions between the protein and the ligand during the simulation during 100 ns (Fig. 9D). The number of hydrogen bonds fluctuates over time, generally ranging between 0 and 3, with occasional peaks reaching up to 4 hydrogen bonds. These fluctuations reflect the dynamic nature of protein–ligand interactions, where hydrogen bonds are continuously formed and broken due to thermal motion and conformational flexibility. Despite this variability, the frequent presence of 1–2 hydrogen bonds throughout the simulation indicates that stable interactions are consistently maintained between the ligand and key residues within the binding pocket. The persistence of these interactions suggests that hydrogen bonding plays a significant role in stabilizing the complex. Notably, higher hydrogen bond counts observed in the early to mid stages of the simulation (approximately 0–60 ns) may indicate stronger initial binding interactions, while the slight reduction in later stages could reflect conformational adaptation of the ligand within the active site. Importantly, the absence of prolonged periods without hydrogen bonds suggests that the ligand remains engaged within the binding pocket and does not dissociate.

##### Solvent accessible surface area (SASA) analysis

The SASA was calculated to evaluate the exposure of the protein surface to the solvent and to monitor potential conformational changes during the 100 ns molecular dynamics simulation (Fig. 9E). The SASA profile shows moderate fluctuations throughout the simulation, ranging approximately between ~ 138 and 150 nm². An initial adjustment phase is observed during the early stage of the trajectory (0–10 ns), followed by a relatively stable regime where SASA values oscillate around an average of ~ 142–145 nm². These fluctuations are indicative of normal protein breathing motions and minor conformational rearrangements in solvent-exposed regions. A slight decrease in SASA observed between ~ 70 and 85 ns suggests a transient increase in protein compactness, potentially associated with tighter packing of secondary structural elements or enhanced ligand accommodation within the binding pocket. Conversely, subsequent increases in SASA indicate reversible conformational flexibility rather than permanent structural changes.

##### Total energy analysis

The total energy of the system was monitored to assess the thermodynamic stability and equilibration of the protein-ligand complex during the simulation (Fig. 9F). The energy profile remains consistently stable over the entire 100 ns trajectory, fluctuating within a narrow range around approximately − 4 × 10⁵ kJ/mol. These small fluctuations are expected due to thermal motion and do not indicate any instability in the system. Importantly, no significant drift or abrupt changes in total energy are observed, suggesting that the system is well-equilibrated and has reached a stable thermodynamic state. The stability of the total energy throughout the simulation confirms that the molecular dynamics protocol was properly conducted, with no artifacts such as system divergence or numerical instability. This behavior further validates the reliability of the simulation results and supports the structural observations obtained from RMSD, RMSF, and Rg analyses.

## Conclusion

This study used a combined quantum and in silico approach to identify the pharmacological candidate 1,2,4-oxadiazolidine (P1_3a), produced via the 32CA reaction between nitrone 1a and carbodiimide 2a.

Analysis using a series of quantum methods, including topological ELF analysis of the reactants, revealed that nitrone 1a is classified as a zw-type dipole, as reported in the literature. CDFT analysis further indicated that dipole 2a is a strong nucleophile, with the chemical potential supporting electron transfer from 1a to 2a, consistent with GEDT transfer in TS transition states. The results suggest an asynchronous, one-step polar mechanism that favors regioselectivity, leading to the formation of an ortho product (P1_3a), which is more thermodynamically stable-especially in the solvent medium (acetonitrile)-and significantly lowers activation barriers.

From a pharmacological perspective, ELF/ESP analysis and molecular docking show that compound P1_3a strongly binds to the inactive domain of EGFR, even outperforming the reference drug Erlotinib in binding energy. These interactions are supported by electrostatic analysis and the presence of hydrogen bonds and hydrophobic interactions. Overall, the ADMET assessment confirms P1_3a’s promising pharmacokinetic properties, including good oral absorption, permeability, no toxicity (hepatic, cardiac, mutagenic), and better tolerability compared to Erlotinib.

These findings suggest that P1_3a, due to its chemical stability and selective reactivity, holds significant potential as a promising computational lead for the inactive EGFR domain. Additional in vitro and in vivo research is necessary to advance new, specific anti-cancer treatments.

## Supplementary Information

Below is the link to the electronic supplementary material.

Supplementary Material 1

## Data Availability

The data used in this work is provided in the manuscript and in the supporting information.
