# Supplementary material for: Computational study of the mechanism and selectivity of [3 + 2] cycloaddition reactions between nitrone and carbodiimide leading to the formation of anticancer 1,2,4-oxadiazolidine compounds from a MEDT perspective
Source: Sci Rep. 2026 Apr 10;16:16104. doi: 10.1038/s41598-026-47611-6 (PMC13199486; doi:10.1038/s41598-026-47611-6)
Supplement: Supplementary file 1 — Supplementary Material 1 [file 41598_2026_47611_MOESM1_ESM.docx]

**Supplementary Material**

**Computational Study of the Mechanism and Selectivity of [3+2] Cycloaddition Reactions Between Nitrone and Carbodiimide Leading to the Formation of Anticancer 1,2,3-Oxadiazolidine Compounds from a MEDT Perspective**

Moulay Driss Mellaoui ^1*^, Mohamed Moussaoui ^2*^, Soukayna Baammi ^2^, Aaziz Jmiai ^1^, Rachid Benhida ^2,3^ , Souad El Issami ^1^, Hanane Zejli ^1^, Rachid Daoud ^2*^

^1^ Applied Physical Chemistry Laboratory, Faculty of Sciences, Ibn Zohr University, B. P. 8106 Cité Dakhla, Agadir, Morocco.

^2^ College of Chemical Sciences and Engineering (CCSE), Chemical and Biochemical Sciences (CBS), Mohammed VI Polytechnic University (UM6P), Benguerir 43150, Morocco,

^3^ Institut de Chimie de Nice (ICN), UMR CNRS 7272, Université Côte d’Azur, Nice, Provence-Alpes- Côte d’Azur, 06108, France

* Corresponding author: Moulay Driss Mellaoui (moulaydriss.mellaoui@edu.uiz.ac.ma)

* Corresponding author: Rachid Daoud (rachid.daoud@um6p.ma)

* Corresponding author: Mohamed Moussaoui ([moussaouimohamed143@gmail.com](mailto:moussaouimohamed143@gmail.com))

**Index**

**Figure S1.** B3LYP-D3/6-311++G(2d,2p) optimized geometries of the TSs. The distances the forming-bond process are given in Å.

**Table S1.**  MPWB1K/6-311++G(2d,2p) Relative energies and thermodynamic properties ∆G, ∆H and ∆S in cal.mol-1.K-1, in the gas phase as well in acetonitrile and ethanol under atmospheric pressure (1atm) and at a temperature of 333 K.

**Table S2.**  WB97XD/6-311++G(2d,2p) Relative energies and thermodynamic properties ∆G, ∆H and ∆S in cal.mol-1.K-1, in the gas phase as well in acetonitrile and ethanol under atmospheric pressure (1atm) and at a temperature of 333 K.

1. **Computational methods**

The theoretical approach conceptual of density functional theory (CDFT) makes it possible to quantitatively evaluate the chemical properties of molecules through global indices such as chemical hardness (η), chemical potential (μ), electrophilicity (ω) and nucleophilicity (N). These descriptors was determined using equations specified in the relevant literature[1–5].

$$\mu=-\frac{\left( E_{LUMO}+E_{HOMO} \right)}{2} (1)$$

$$\eta=\frac{(E_{LUMO}-E_{HOMO})}{2} (2)$$

$$\omega=\frac{\mu^{2}}{2\eta} (3)$$

$$N= E_{HOMO}\left( reactive \right)- E_{HOMO}\left( TCE \right) (4)$$

To predict the polar character of the 32CA reactions, the global electron density transfer (GEDT) [6] at the TSs was calculated as the summation of natural atomic charges [7,8] (q) using the formula :

$$GEDT (f)= (5)$$

where a positive GEDT denotes an electron density flux from the considered framework.

We also performed the electron localization function (ELF) [9,10] topological studies using Multiwfn Version 3.8 (dev) software [11]. The ELF localization domains were represented using UCSF Chimera software [12]. The independent gradient model (IGM) approach [13], supported by the electron density (ED) topology was employed using the corresponding mono determinantal wave functions of the ground state (GS) of the transition structures at the B3LYP-D3/6-31G(d) level using the Multiwfn Version 3.8 (dev) software [11]. The latter allows probing the interactions at the TSs in order to reveal the evolution of the forming bonds. The IGM isosurfaces were visualized using the VMD software [14].

The most successful protein-ligand combination, exhibiting the lowest free binding energy (obtained after the docking molecular), was then subjected to a 100 ns molecular dynamics (MD) simulation using Gromacs 2021 (<https://ftp.gromacs.org/gromacs/gromacs-2021>). At the starting point, the simulation starts by the separation of the protein and the ligand from their respective complex form, in order to generate the individual topology files for the protein and the ligand. Here, we built the protein topology using the parameters implemented in CHARMM-36[15]. The protein topology was constructed with the “charmm36-” force field and the TIP3-Point water model, recommended by CHARMM. The ligand topologies were generated using the CHARMM-based online server - CGenFF server[16].

The protein-ligand complex was regenerated by manually adjusting the residues in the MD.gro and box_sol_ion.gro files in a new complex.gro file. The next step was to solvate the complex in a dodecahedral box using the water model, then neutralize the system with appropriate positive (Na⁺) and negative (Cl⁻) ions. The generated complex-solvent was neutralized by adding 14 Na⁺ ions. To minimize the energy of the generated complex-solvent system, the steepest descent minimization method was used; minimization was performed over a maximum of 50,000 steps. The ligand constraint position index file was generated and its .itp file was incorporated into the main topology file.

Using the leap-frog integrator algorithm, the NVT (constant number of particles, volume, and temperature) and NPT (constant number of particles, pressure, and temperature) ensembles were subjected to 500,000 steps, equivalent to 100 nacoseconds, at a temperature of 300 K.

The resulting balanced system was finally subjected to an MD simulation of 100 ns. The Particle Mesh Ewald (PME) method[17] was used to handle long-range electrostatic interactions, such as Coulomb and Lennard-Jones interactions, with the LINCS algorithm[18]. For covalent bonds, the cutoff value was set at 12 Å.

1. **Mechanistic pathways in the cheletropic cycloaddition reactions of nitrone 1a and carbodiimide 2a**
   1. **Optimization of transition states**

**
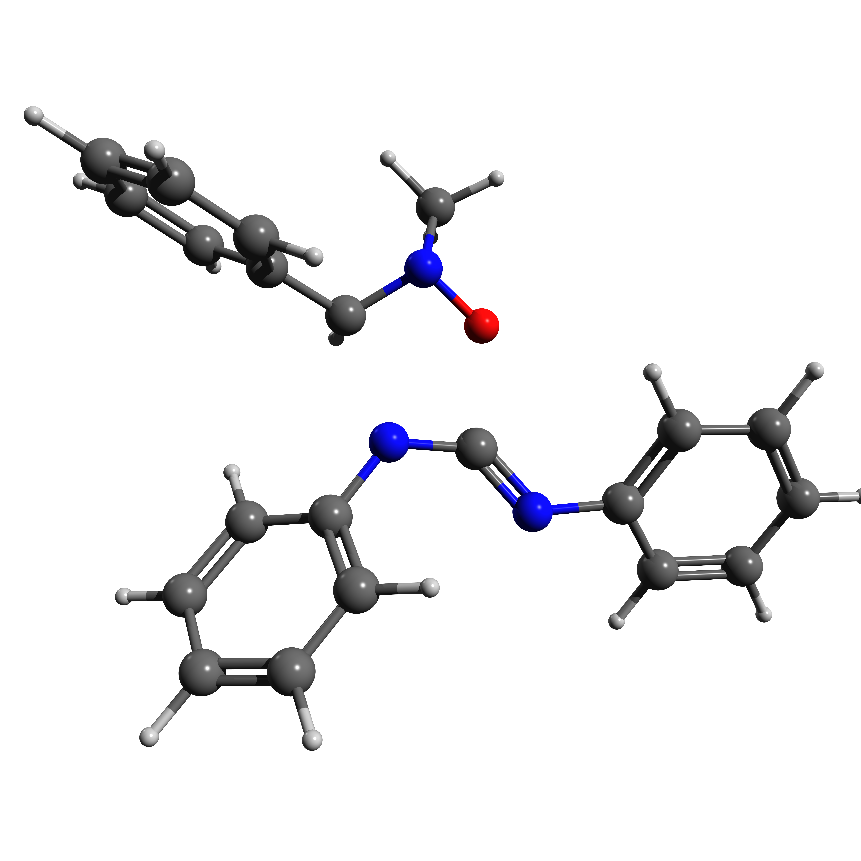
**

**TS1_3a**

**2.37**

**2.15**

**
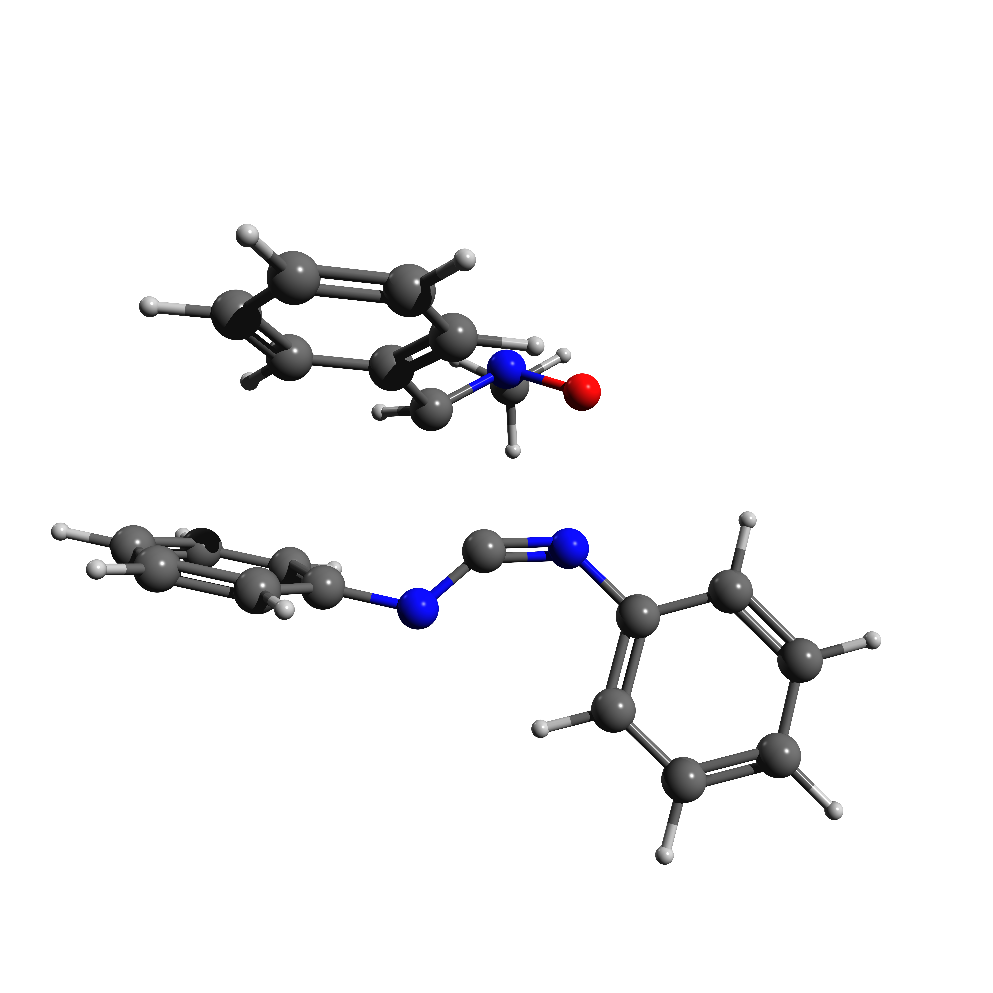
**

**TS2_3a**

**2.24**

**2.33**

**Figure S1.** B3LYP-D3/6-311++G(2d,2p) optimized geometries of the TSs. The distances the forming-bond process are given in Å.

**Table S1.**  MPWB1K/6-311++G(2d,2p) Relative energies and thermodynamic properties ∆G, ∆H and ∆S in cal.mol-1.K-1, in the gas phase as well in acetonitrile under atmospheric pressure (1atm) and at a temperature of 333 K.

| **TS/P** | **Phase** | **∆E** | **∆H** | **∆G** | **∆S** | **GEDT** |
| --- | --- | --- | --- | --- | --- | --- |
| **TS1-3a** | Gas phase | 88.89 | 35.49 | 44.98 | -25.39 | 0.08 |
| **P1-3a** | Gas phase | -16.04 | -62.77 | -55.91 | -17.48 |  |
| **TS2-3a** | Gas phase | 53.49 | 3.83 | 10.45 | -16.77 | 0.09 |
| **P2-3a** | Gas phase | -9.08 | -42.35 | -36.13 | -18.99 |  |
| **TS1-3a** | Acetonitrile | 57.60 | 74.04 | 91.26 | -57.93 | 0.14 |
| **P1-3a** | Acetonitrile | -45.71 | -22.60 | -7.06 | -52.89 |  |
| **TS2-3a** | Acetonitrile | 23.47 | 43.59 | 58.12 | -49.85 | 0.15 |
| **P2-3a** | Acetonitrile | -43.23 | -16.56 | -6.84 | -29.48 |  |

**Table S2.** WB97XD/6-311++G(2d,2p) Relative energies and thermodynamic properties ∆G, ∆H and ∆S in cal.mol-1.K-1, in the gas phase and Acetonitrile under atmospheric pressure (1atm) and at a temperature of 333 K.

| **TS/P** | **Phase** | **∆E** | **∆H** | **∆G** | **∆S** | **GEDT** |
| --- | --- | --- | --- | --- | --- | --- |
| **TS1-3a** | Gas phase | 66.88 | 65.36 | 81.04 | -49.06 | 0.08 |
| **P1-3a** | Gas phase | -28.86 | -25.34 | -8.30 | -53.14 |  |
| **TS2-3a** | Gas phase | 33.57 | 33.35 | 51.11 | -55.29 | 0.09 |
| **P2-3a** | Gas phase | -5.22 | -21.16 | -4.72 | -48.52 |  |
| **TS1-3a** | Acetonitrile | 70.16 | 66.89 | 79.94 | -39.16 | 0.14 |
| **P1-3a** | Acetonitrile | -24.45 | -22.34 | -9.02 | -39.96 |  |
| **TS2-3a** | Acetonitrile | 47.58 | 44.40 | 64.29 | -59.67 | 0.15 |
| **P2-3a** | Acetonitrile | -21.26 | -3.95 | -8.06 | -37.81 |  |

- 1. **Bonding Evolution Theory (BET) at the formation P1_3a**

The Bonding Evolution Theory (BET) is a conceptual approach based on the analysis of electron densities in chemical reactions, in particular through the structures of intermediates and transition states [47,48]. It helps to understand the path of chemical bonding, from initial to formation, by investigating bond distances, bassin populations (V), and global electron transfer charges (GEDT). In the study of cycloaddition 1,3-dipolar reaction between nitrone 1a and carbodiimide 2a, BET makes it possible to follow the transformation of the complex MC-I to the cycloadduct P1-3a in eleven steps (S0 to S8). This analysis highlights the progressive structural changes, in particular variations in critical interatomic distances (d(O1-C5) and d(C3-N4)), relative energy (ΔE), and electronic delocalization involved in the formation of the final 1,2,4-oxadiazolidine ring. The values from this analysis are abstracted in **Table S3**, and the detailed reaction mechanism is shown in **Scheme 3**.

**Table S3.** ELF valence basin populations, distances of the forming bonds, and relative electronic energies of the IRC structures **MC-I**– **P1-3a** defining the 8 phases characterizing the molecular mechanism of the 32CA reaction of nitrone (**a**) with carbodiimide 2a to form the product 1,2,4-oxadiazolidine

| *Phases* | *I* | | *II* | | *III* | *IV* | *V* | *VI* | *VII* | *VI* | |
| --- | --- | --- | --- | --- | --- | --- | --- | --- | --- | --- | --- |
| Structures | **MC-I** | **S0** | **S1** | **S2** | **S3** | **S4** | **S5** | **S6** | **S7** | **S8** | **P1-3a** |
| d(O1-C5) in Å | 3.09 | 3.04 | 1.95 | 2.15 | 1.59 | 1.54 | 1.50 | 1.45 | 1.41 | 1.39 | 1.35 |
| d(C3-N4) in Å | 3.13 | 3.14 | 2.52 | 2.37 | 2.35 | 2.27 | 2.20 | 2.01 | 1.78 | 1.53 | 1.29 |
| ΔE (kcal.mol^-1^) | 0.00 | 5.47 | 12.83 | 89.64 | 39.68 | 20.43 | 13.55 | 10.78 | 4.13 | -9.05 | -18.98 |
| GEDT | 0.01 | 0.02 | 0.33 | 0.17 | 0.43 | 0.39 | 0.43 | 0.33 | 0.16 | 0.01 | -0.13 |
| V(O1) | 2.92 | 2.90 | 2.91 | 2.99 | 2.58 | 2.43 | 2.40 | 2.32 | 2.27 | 2.23 | 2.19 |
| V'(O1) | 3.05 | 3.07 | 3.03 | 2.84 | 2.83 | 2.80 | 2.78 | 2.73 | 2.72 | 2.72 | 2.72 |
| V(O1-N2) | 1.37 | 1.36 | 1.23 | 1.23 | 1.08 | 1.05 | 1.02 | 0.96 | 0.94 | 0.92 | 0.90 |
| V(N2-C3) | 2.51 | 2.49 | 3.86 | 2.08 | 2.73 | 2.64 | 2.57 | 2.29 | 2.04 | 1.92 | 1.81 |
| V'(N2-C3) | 1.26 | 1.28 |  |  |  |  |  |  |  |  |  |
| V(N4) | 2.82 | 2.81 | 3.02 | 3.22 | 3.11 | 3.15 | 3.17 | 1.50 | 0.91 | 1.38 | 1.84 |
| V(N4-C5) | 3.14 | 3.13 | 2.93 | 2.45 | 2.62 | 2.54 | 2.47 | 2.31 | 2.13 | 2.02 | 1.92 |
| V(C5) |  |  |  |  |  | 1.02 |  |  |  |  |  |
| V(N2) |  |  |  |  | 1.39 | 1.53 | 1.66 | 1.94 | 2.18 | 2.30 | 2.41 |
| V'(N4) |  |  |  |  |  |  |  | 1.74 |  |  |  |
| V(C3) |  |  |  |  |  |  |  |  | 0.18 |  |  |
| V(O1,C5) |  |  |  |  |  |  | 1.10 | 1.27 | 1.32 | 1.45 | 1.58 |
| V(C3,N4) |  |  |  |  |  |  |  |  |  | 1.45 | 1.46 |


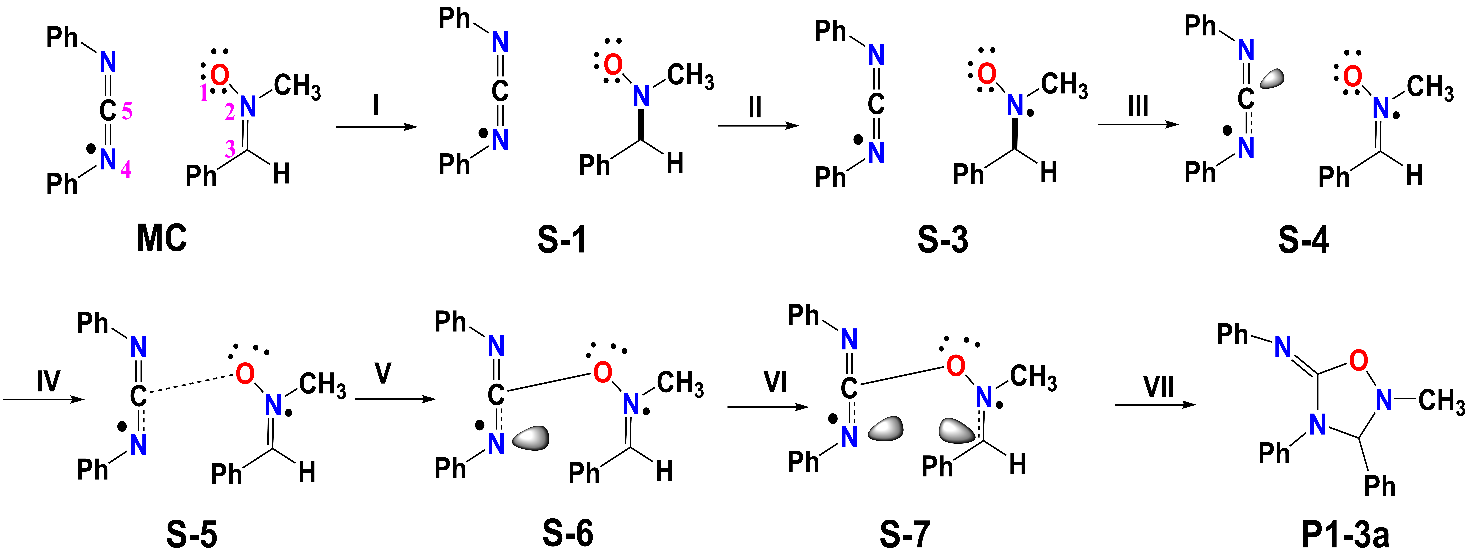


**Scheme S1.**  Mechanistic illustration of the 32CA reaction between nitrone (1a) and

carbodiimide (2a), inspired by Lewis structures based on ELF analysis.

***TS1-3b* ( -377.50 cm^-1^)**

0 1

O -0.555135000 -1.909303000 -0.490779000

N 0.658428000 -2.244275000 -0.210932000

C 1.450213000 -1.198602000 -0.757325000

N 0.069127000 0.584325000 -0.034193000

C -1.165765000 0.106889000 -0.046162000

C 0.881481000 -3.567525000 -0.775487000

H 0.732990000 -3.569779000 -1.859184000

H 1.901335000 -3.864700000 -0.540570000

H 0.189183000 -4.262609000 -0.309369000

C 2.814063000 -1.153303000 -0.177129000

C 3.931492000 -1.336095000 -0.991626000

C 5.206949000 -1.330335000 -0.442281000

C 5.370899000 -1.128604000 0.924859000

C 4.258412000 -0.939534000 1.740646000

C 2.983297000 -0.954474000 1.193542000

H 3.799489000 -1.481733000 -2.055983000

H 6.069352000 -1.476739000 -1.077011000

H 6.363338000 -1.114133000 1.353009000

H 4.387183000 -0.777481000 2.801395000

H 2.111950000 -0.805000000 1.813748000

N -2.298114000 0.645914000 0.012472000

C -3.515988000 -0.042902000 0.083098000

C -4.601286000 0.472372000 -0.632343000

C -5.846303000 -0.137848000 -0.562036000

C -6.035933000 -1.261725000 0.236145000

C -4.963126000 -1.767917000 0.964219000

C -3.712828000 -1.169014000 0.891848000

H -4.447708000 1.353583000 -1.238560000

H -6.672555000 0.269960000 -1.128263000

H -7.006939000 -1.732606000 0.295742000

H -5.100153000 -2.635689000 1.595269000

H -2.883579000 -1.567211000 1.456954000

C 0.406561000 1.950305000 -0.068375000

C -0.295172000 2.877386000 0.706831000

C 0.067802000 4.216243000 0.678231000

C 1.133450000 4.648183000 -0.106059000

C 1.833354000 3.723399000 -0.871461000

C 1.472698000 2.381752000 -0.859166000

H -1.125558000 2.544520000 1.307857000

H -0.484552000 4.925617000 1.278769000

H 1.414235000 5.691594000 -0.118990000

H 2.663052000 4.043299000 -1.486359000

H 2.021914000 1.674142000 -1.460043000

H 1.456391000 -1.174255000 -1.853425000

***TS2_3b* ( -349.39 cm^-1^)**

0 1

O 1.047406000 -2.187467000 1.204001000

N -0.024822000 -1.874862000 1.862647000

C -0.819527000 -0.938395000 1.145333000

N 1.832225000 -0.180545000 0.322569000

C 0.740918000 0.399274000 0.250265000

C 0.320690000 -1.454578000 3.234685000

H 0.876781000 -0.516836000 3.216684000

H -0.596412000 -1.339539000 3.807410000

H 0.926935000 -2.236657000 3.679567000

H -1.428644000 -0.393952000 1.858934000

C -1.670386000 -1.437689000 0.024819000

C -2.970206000 -0.946088000 -0.100042000

C -3.779838000 -1.360984000 -1.148141000

C -3.298458000 -2.270482000 -2.083184000

C -2.000837000 -2.762002000 -1.965392000

C -1.185473000 -2.344650000 -0.922778000

H -3.340543000 -0.222634000 0.611621000

H -4.783204000 -0.968986000 -1.235623000

H -3.927952000 -2.593040000 -2.900715000

H -1.621461000 -3.467670000 -2.691069000

H -0.177994000 -2.721852000 -0.833972000

N 0.226452000 1.482320000 -0.175426000

C -1.107641000 1.871279000 -0.007742000

C -1.962896000 1.912698000 -1.112444000

C -3.286408000 2.301305000 -0.963188000

C -3.777310000 2.672701000 0.285538000

C -2.922813000 2.666017000 1.383963000

C -1.597177000 2.274687000 1.239652000

H -1.578699000 1.609933000 -2.075448000

H -3.939918000 2.308330000 -1.824542000

H -4.808273000 2.976425000 0.398827000

H -3.286716000 2.972931000 2.355211000

H -0.926345000 2.288032000 2.087701000

C 3.115809000 0.146141000 -0.144188000

C 4.153349000 -0.778380000 -0.055316000

C 5.417305000 -0.436362000 -0.521275000

C 5.655255000 0.816383000 -1.076094000

C 4.611119000 1.730095000 -1.158173000

C 3.341566000 1.410060000 -0.689296000

H 3.967845000 -1.752145000 0.370136000

H 6.218189000 -1.159518000 -0.450623000

H 6.639652000 1.076381000 -1.437931000

H 4.778981000 2.708359000 -1.587674000

H 2.529289000 2.117586000 -0.751064000

***Product P1_3a***

0 1

O 1.090573000 -1.540041000 0.390654000

N -0.272606000 -2.065376000 0.236327000

C -1.105117000 -0.931412000 0.671698000

N -0.309543000 0.176787000 0.161581000

C 1.022984000 -0.197417000 0.154592000

C -0.363964000 -3.219491000 1.111800000

H -0.152903000 -2.972223000 2.157757000

H -1.377182000 -3.607788000 1.022536000

H 0.333332000 -3.977752000 0.766895000

C -2.487123000 -1.046800000 0.086892000

C -3.571012000 -1.314098000 0.915224000

C -4.848269000 -1.449133000 0.380194000

C -5.041698000 -1.310945000 -0.988731000

C -3.956692000 -1.043365000 -1.820618000

C -2.683035000 -0.915493000 -1.285728000

H -3.418192000 -1.410230000 1.982575000

H -5.686829000 -1.655448000 1.030135000

H -6.033323000 -1.408536000 -1.407784000

H -4.106223000 -0.933756000 -2.885445000

H -1.836206000 -0.708395000 -1.923399000

N 2.011783000 0.573441000 0.005459000

C 3.335887000 0.116907000 -0.091045000

C 4.324201000 0.844854000 0.578858000

C 5.660217000 0.479968000 0.480790000

C 6.038155000 -0.605505000 -0.303274000

C 5.061464000 -1.321720000 -0.988737000

C 3.722244000 -0.969834000 -0.885685000

H 4.023080000 1.696390000 1.172089000

H 6.409162000 1.050664000 1.012789000

H 7.078937000 -0.884965000 -0.385562000

H 5.343290000 -2.161203000 -1.609894000

H 2.971216000 -1.531055000 -1.421309000

C -0.766807000 1.513450000 0.149925000

C -0.195470000 2.443722000 -0.720907000

C -0.668174000 3.747424000 -0.744503000

C -1.717401000 4.139917000 0.081624000

C -2.288337000 3.211223000 0.941669000

C -1.815173000 1.905031000 0.982996000

H 0.620101000 2.143614000 -1.356815000

H -0.215750000 4.459609000 -1.420308000

H -2.084912000 5.155667000 0.052849000

H -3.104079000 3.498864000 1.590013000

H -2.268838000 1.199372000 1.660302000

H -1.167975000 -0.890152000 1.771579000

***Product P2_3a***

1. 1

O 1.654957000 1.467721000 -1.074660000

N 0.486451000 1.575931000 -1.879404000

C -0.502779000 0.771424000 -1.148026000

N 1.652241000 0.099730000 -0.589953000

C 0.353747000 -0.320239000 -0.482745000

C 0.785554000 1.034792000 -3.207472000

H 1.063961000 -0.023533000 -3.186474000

H -0.103428000 1.166182000 -3.821575000

H 1.596397000 1.615290000 -3.637416000

H -1.184127000 0.320601000 -1.862508000

C -1.303183000 1.543367000 -0.123131000

C -2.677607000 1.336892000 -0.038891000

C -3.433260000 2.005738000 0.916912000

C -2.817911000 2.887501000 1.797134000

C -1.444116000 3.096998000 1.716547000

C -0.687556000 2.426236000 0.764392000

H -3.156989000 0.636393000 -0.708794000

H -4.498762000 1.833263000 0.974322000

H -3.403218000 3.408692000 2.541768000

H -0.960306000 3.782766000 2.398016000

H 0.377533000 2.592394000 0.703057000

N -0.014863000 -1.410975000 0.059483000

C -1.371490000 -1.780392000 0.029396000

C -2.105714000 -1.795231000 1.217868000

C -3.444356000 -2.159373000 1.209348000

C -4.069164000 -2.532051000 0.021949000

C -3.333946000 -2.549386000 -1.158466000

C -1.993265000 -2.181291000 -1.156981000

H -1.618872000 -1.492826000 2.133581000

H -4.004651000 -2.147493000 2.134061000

H -5.111468000 -2.817255000 0.019559000

H -3.802101000 -2.855599000 -2.084087000

H -1.415732000 -2.211609000 -2.070857000

C 2.823986000 -0.285864000 0.070441000

C 3.964114000 0.519420000 -0.029797000

C 5.136628000 0.129745000 0.601882000

C 5.193568000 -1.053171000 1.330251000

C 4.056033000 -1.847574000 1.422085000

C 2.869988000 -1.478589000 0.802067000

H 3.925193000 1.437575000 -0.591822000

H 6.011102000 0.760264000 0.521300000

H 6.110314000 -1.351240000 1.818245000

H 4.084606000 -2.770518000 1.984489000

H 1.988439000 -2.091622000 0.872957000

**References**

[1] S. - Liu, - Electrophilicity Index, 121 (1999). - https://pubs.acs.org/doi/10.1021/ja983494x.

[2] P. - Pérez, - The nucleophilicity N index in organic chemistry, 9 (2011). - http://xlink.rsc.org/?DOI=c1ob05856h.

[3] M.D. Mellaoui, K. Abbiche, N. Acharjee, H. Mohammad-Salim, A. Imjjad, R. Boutiddar, K. Marakchi, S. El Issami, H. Zejli, Unveiling the mechanism and selectivity of the [3 + 2] cycloaddition reactions of nitrone with acetylene derivatives leading to anticancer 4-isoxazoline derivatives from the MEDT perspective, Computational and Theoretical Chemistry 1237 (2024) 114619. https://doi.org/10.1016/j.comptc.2024.114619.

[4] P. - Ayers, - Conceptual density functional theory: status, prospects, issues, 139 (2020). - http://link.springer.com/10.1007/s00214-020-2546-7.

[5] W. - Langenaeker, - Conceptual Density Functional Theory, 103 (2003). - https://pubs.acs.org/doi/10.1021/cr990029p.

[6] L.R. Domingo, A new C–C bond formation model based on the quantum chemical topology of electron density, RSC Adv. 4 (2014) 32415–32428. https://doi.org/10.1039/C4RA04280H.

[7] A.E. Reed, R.B. Weinstock, F. Weinhold, Natural population analysis, The Journal of Chemical Physics 83 (1985) 735–746.

[8] A.E. Reed, L.A. Curtiss, F. Weinhold, Intermolecular interactions from a natural bond orbital, donor-acceptor viewpoint, Chemical Reviews 88 (1988) 899–926.

[9] A.D. Becke, K.E. Edgecombe, A simple measure of electron localization in atomic and molecular systems, The Journal of Chemical Physics 92 (1990) 5397–5403. https://doi.org/10.1063/1.458517.

[10] B. Silvi, A. Savin, Classification of chemical bonds based on topological analysis of electron localization functions, Nature 371 (1994) 683–686. https://doi.org/10.1038/371683a0.

[11] T. Lu, F. Chen, Multiwfn: A multifunctional wavefunction analyzer, Journal of Computational Chemistry 33 (2012) 580–592. https://doi.org/10.1002/jcc.22885.

[12] E.F. Pettersen, T.D. Goddard, C.C. Huang, G.S. Couch, D.M. Greenblatt, E.C. Meng, T.E. Ferrin, UCSF Chimera—A visualization system for exploratory research and analysis, Journal of Computational Chemistry 25 (2004) 1605–1612. https://doi.org/10.1002/jcc.20084.

[13] C. Lefebvre, H. Khartabil, J.-C. Boisson, J. Contreras-García, J.-P. Piquemal, E. Hénon, The Independent Gradient Model: A New Approach for Probing Strong and Weak Interactions in Molecules from Wave Function Calculations, ChemPhysChem 19 (2018) 724–735. https://doi.org/10.1002/cphc.201701325.

[14] W. Humphrey, A. Dalke, K. Schulten, VMD: Visual molecular dynamics, Journal of Molecular Graphics 14 (1996) 33–38. https://doi.org/10.1016/0263-7855(96)00018-5.

[15] R.B. Best, X. Zhu, J. Shim, P.E.M. Lopes, J. Mittal, M. Feig, A.D. MacKerell, Optimization of the Additive CHARMM All-Atom Protein Force Field Targeting Improved Sampling of the Backbone ϕ, ψ and Side-Chain χ1 and χ2 Dihedral Angles, J. Chem. Theory Comput. 8 (2012) 3257–3273. https://doi.org/10.1021/ct300400x.

[16] K. Vanommeslaeghe, E. Hatcher, C. Acharya, S. Kundu, S. Zhong, J. Shim, E. Darian, O. Guvench, P. Lopes, I. Vorobyov, A.D. Mackerell, CHARMM general force field: A force field for drug‐like molecules compatible with the CHARMM all‐atom additive biological force fields, J Comput Chem 31 (2010) 671–690. https://doi.org/10.1002/jcc.21367.

[17] H.G. Petersen, Accuracy and efficiency of the particle mesh Ewald method, The Journal of Chemical Physics 103 (1995) 3668–3679. https://doi.org/10.1063/1.470043.

[18] B. Hess, H. Bekker, H.J.C. Berendsen, J.G.E.M. Fraaije, LINCS: A linear constraint solver for molecular simulations, J. Comput. Chem. 18 (1997) 1463–1472. https://doi.org/10.1002/(SICI)1096-987X(199709)18:12<1463::AID-JCC4>3.0.CO;2-H.
